# Long-Term Risk of Parkinson’s Disease Following Irritable Bowel Syndrome: A Nationwide Population-Based Cohort Study

**DOI:** 10.3390/healthcare14101329

**Published:** 2026-05-13

**Authors:** Youngoh Bae, Seondeok Seo, Sang Ryong Jeon, Jae June Dong, Seung Won Lee, Hohyun Jung

**Affiliations:** 1 Department of Precision Medicine, Sungkyunkwan University School of Medicine, Suwon 16419, Republic of Korea; yobae05@gmail.com; 2 Department of Neurological Surgery, Asan Medical Center, University of Ulsan College of Medicine, Seoul 05505, Republic of Korea; srjeon190@gmail.com; 3 Department of Artificial Intelligence, Sungkyunkwan University, Suwon 16419, Republic of Korea; pusdseo0506@gmail.com; 4 Department of Family Medicine, Kangbuk Samsung Hospital, Sungkyunkwan University School of Medicine, Seoul 03181, Republic of Korea; jaejune.dong@samsung.com; 5 Department of Statistics, Sungshin Women’s University, Seoul 02844, Republic of Korea; 6 Center for Data Science, Sungshin Women’s University, Seoul 02844, Republic of Korea

**Keywords:** irritable bowel syndrome, Parkinson’s disease, gut–brain axis, nationwide cohort study

## Abstract

**Background/Objectives**: Irritable bowel syndrome (IBS) is a common functional gastrointestinal disorder linked to gut–brain axis dysregulation. Gastrointestinal dysfunction has been implicated in the prodromal phase of Parkinson’s disease (PD), but long-term population-based evidence remains limited. **Methods:** Using the Korean National Health Insurance Service database (2012–2023), we conducted a nationwide matched cohort study including 142,668 patients with IBS and 285,336 matched individssuals without IBS. IBS was defined by at least two ICD-10 diagnoses with a three-year washout period to reduce reverse causation. Participants were followed for up to nine years for incident PD. Incidence rate ratios (IRRs), Kaplan–Meier analyses, and multivariable Cox models were applied. **Results:** IBS was associated with a higher incidence of PD (IRR, 1.24; 95% CI, 1.14–1.36). The magnitude and statistical significance of the association varied across age- and sex-stratified analyses, with the highest crude relative risk observed among women younger than 60 years. In fully adjusted Cox models, IBS remained significantly associated with PD in the overall population (hazard ratio, 1.38; 95% CI, 1.18–1.62), with statistical significance retained particularly among women in subgroup analyses. Sensitivity analyses using alternative definitions yielded consistent results. **Conclusions:** In this nationwide cohort, IBS was associated with an increased long-term risk of PD after adjustment for measured covariates. Given the observational claims-based design, these findings should be interpreted cautiously as hypothesis-generating epidemiological evidence requiring further validation.

## 1. Introduction

Irritable bowel syndrome (IBS) is a common functional gastrointestinal disorder characterized by recurrent abdominal pain or discomfort accompanied by alterations in bowel habits, without identifiable structural or biochemical abnormalities. Diagnosis is primarily based on the Rome criteria, and IBS is classified into several subtypes, including diarrhea-predominant, constipation-predominant, and mixed forms [1]. IBS is prevalent across all age groups, with reported prevalence rates of approximately 9.7% among middle-aged adults and 7.5% among older adults [2]. Given its association with reduced quality of life, increased healthcare utilization, and productivity loss, IBS is a clinically significant condition whose long-term health implications warrant further investigation [3].

IBS may represent a relevant gastrointestinal condition for investigating the long-term epidemiological association between chronic gut-related dysfunction and Parkinson’s disease (PD). IBS has been associated with gut–brain axis dysregulation, gut microbiota dysbiosis, impaired intestinal barrier function, low-grade mucosal immune activation, and inflammatory responses [4,5]. Gastrointestinal dysfunction, including constipation, has also been recognized as a frequent prodromal non-motor manifestation of PD [6]. Moreover, the “gut-first” hypothesis proposes that α-synuclein pathology may originate in the enteric nervous system and subsequently propagate to the central nervous system, potentially through vagal and other gut–brain pathways [7,8]. Collectively, these observations provide biological rationale for investigating whether IBS is associated with subsequent PD risk, without implying that IBS is a clinically established risk marker for PD.

Previous epidemiological studies have reported an association between IBS and an increased risk of PD compared with the general population. A meta-analysis synthesizing multiple observational studies reported an approximately 1.5-fold increased risk of PD among patients with IBS [9]. One representative cohort study using the Finnish nationwide health registry reported an increased risk of PD following an IBS diagnosis; however, the excess risk was largely confined to the first two years after diagnosis and was not statistically significant during extended follow-up [10]. This pattern limits the interpretation of IBS as a long-term antecedent condition for PD and raises concerns regarding reverse causation, prodromal detection, or surveillance bias. Therefore, further studies with stricter exposure definitions, sufficient washout periods, and extended follow-up are needed to clarify the temporal association between IBS and subsequent PD risk.

This large-scale, population-based cohort study used data from the Korean National Health Insurance Service (NHIS). Approximately two million individuals were analyzed, applying a three-year washout period and up to nine years of follow-up to assess the risk of PD after IBS. Case and reference cohorts were constructed using exact matching on sex, age, and health screening year to ensure baseline comparability. Outcomes were defined as PD and PD-related disorders to evaluate the consistency and robustness of the associations. The extended follow-up enabled a clear assessment of the temporal relationship between IBS and subsequent PD risk.

## 2. Materials and Methods

### 2.1. Data Sources

This study aimed to evaluate the association between IBS and subsequent PD risk. The analysis utilized health insurance claims and health screening data from 2012 to 2023 provided by the NHIS, a single-payer, population-based public healthcare system covering the entire Korean population. The study population was drawn from an approximately two-million-person sample cohort. The NHIS database comprises large-scale administrative data, including healthcare utilization and reimbursement records, International Classification of Diseases, 10th Revision (ICD-10) based diagnostic codes, procedures and prescription information, sociodemographic variables, and results from standardized health examinations [11]. These data encompass diverse demographic characteristics such as sex, age, and income level, and the NHIS sample cohort is known to achieve national representativeness through stratified random sampling based on approximately 1476 strata defined by key demographic variables [11]. NHIS claims records and health screening data allow long-term patient follow-up and enable assessment of subsequent PD risk after IBS in a large-scale population-based setting, with adjustment for demographic, lifestyle, and clinical covariates [12].

Ethical approval for this study was waived by the Institutional Review Board of Sungshin Women’s University (SSWUIRB-2025-105). Informed consent was also waived because the NHIS data were fully anonymized and contained no personally identifiable information.

### 2.2. Definition of Exposure and Outcome

In this study, IBS and PD were defined using ICD-10 codes K58 and G20, respectively, based on prior claims-based research [13] (Supplementary Table S1). Unlike previous studies that defined IBS using at least one recorded K58 diagnosis, the present study required at least two recorded IBS diagnoses. This definition was intended to reduce misclassification from single, rule-out, or transient diagnostic coding.

### 2.3. Case and Control Group Selection

**Figure 1:**
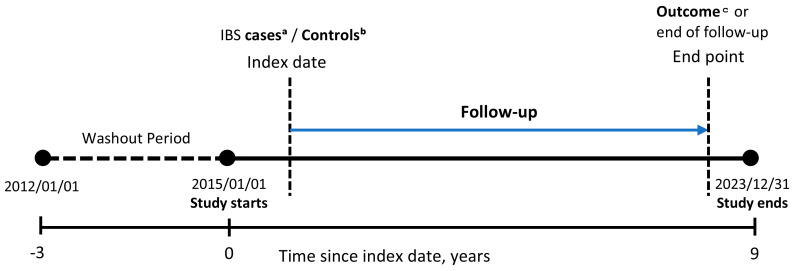
Study overview and ascertainment of exposure, covariates, and outcome. ᵃ IBS cases: Individuals with ≥2 diagnoses of ICD-10 code K58 were included, and the first diagnosis date was assigned as the index date; ᵇ Controls: No diagnosis of IBS (ICD-10; K58) during the washout and follow-up periods; ᶜ Outcome: First occurrence of idiopathic Parkinson’s disease, defined using ICD-10 code G20, after the index date.

To minimize reverse causation, this study designated the period from 2012 to 2014 as a washout period. This approach was intended to exclude pre-existing disease histories or prodromal symptom progression that could influence the observed associations, and the methodological importance of implementing a washout period has been emphasized in prior research [14]. The observation period was subsequently defined as 2015–2023 to more accurately estimate the true risk of PD following IBS (Figure 1). Follow-up was terminated at the earliest occurrence of (1) the first diagnosis of PD, (2) death, or (3) the end of the study period (31 December 2023). The mean follow-up duration and corresponding standard deviation for each case cohort and its matched reference cohort are presented in Supplementary Table S2.

**Figure 2:**
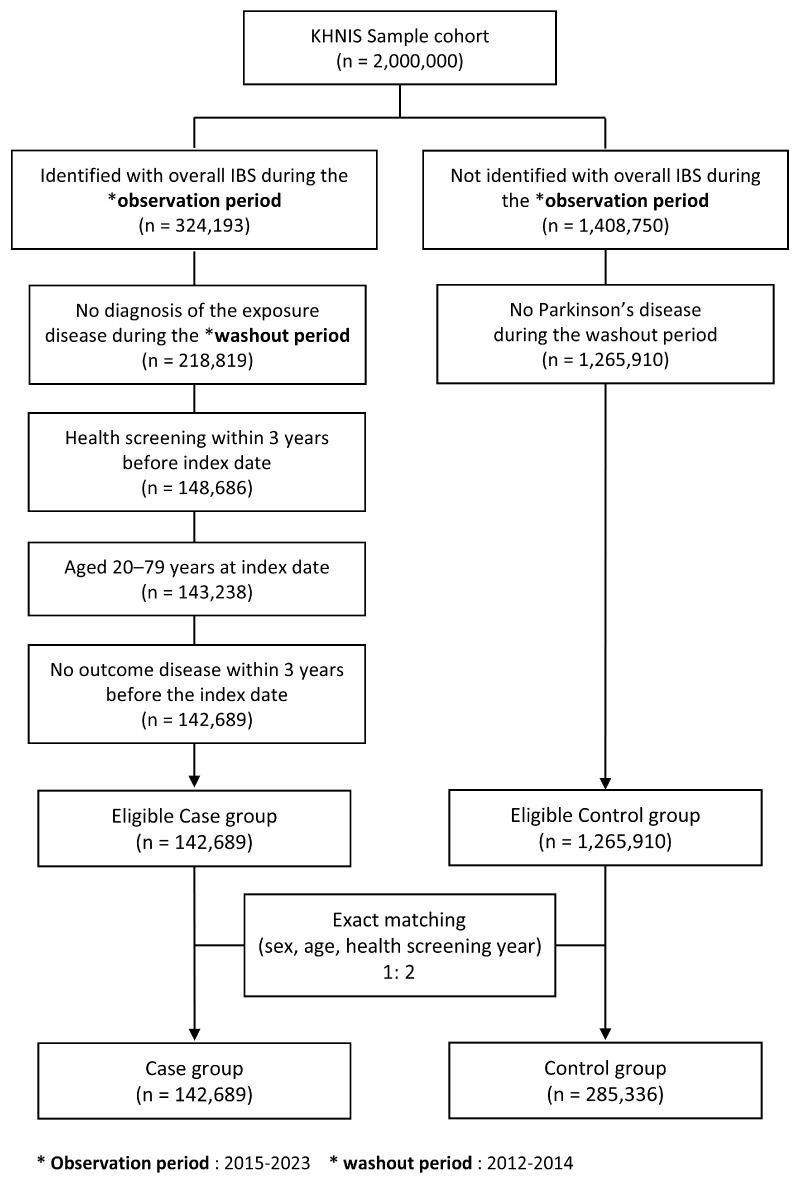
Flowchart of IBS case cohort and matched reference cohort selection.

Individuals were excluded from the case cohort if they met any of the following criteria: (1) a diagnosis of IBS during the washout period; (2) absence of health screening records within three years prior to the index date; (3) age younger than 20 years or older than 80 years at the index date; (4) a prior diagnosis of PD or parkinsonism-related disorders (ICD-10 codes G20-G23) during the washout period, to ensure identification of incident cases; or (5) a diagnosis of major neurodegenerative diseases, structural central nervous system disorders, or organic gastrointestinal diseases that could compromise the validity of the IBS diagnosis (Figure 2). This age range was selected to include adult patients with sufficient follow-up while reducing diagnostic uncertainty and clinical heterogeneity at the extremes of age. The detailed ICD-10 codes for these conditions are provided in Supplementary Table S3.

The reference cohort was constructed by exact matching to the case cohort at a 1:2 ratio based on sex, age, and health screening year. Exact matching by age and sex was applied to minimize confounding from these major determinants of PD incidence and to preserve comparability between cohorts across the broad adult age range. Because the standardized differences for these matching variables were all below 0.1, indicating adequate balance between groups, propensity score matching was not applied (Table 1). The same matching criteria were used in the sensitivity analyses, which likewise demonstrated standardized differences below 0.1 across all matching variables, confirming sufficient group balance (Supplementary Table S4).

### 2.4. Variables

The analyses incorporated key health screening variables, including sex, age, smoking status, alcohol consumption, height, weight, body mass index (BMI), total cholesterol, systolic and diastolic blood pressure, fasting glucose, and income level. Each variable was extracted from the health screening record closest to the index date. Height and weight were treated as continuous variables, whereas the remaining indicators were categorized according to clinically relevant thresholds for analytical purposes.

### 2.5. Baseline Characteristics

Baseline characteristics of the case and reference cohorts were compared using appropriate statistical methods according to variable type. Continuous variables were evaluated using Student’s *t* test, while categorical variables were compared using Fisher’s exact test or Pearson’s χ^2^ test, as appropriate. In addition, standardized differences were calculated to assess covariate balance, with values below 0.1 considered indicative of negligible differences between groups.

### 2.6. Statistical Analysis

To compare the risk of PD following an IBS diagnosis, crude incidence rates (IRs) per 1000 person-years and incidence rate ratios (IRRs) were calculated. Time-to-event cumulative incidence was illustrated using Kaplan–Meier curves, and differences between the IBS and reference cohorts were assessed with the log-rank test. In the Kaplan–Meier and Cox proportional hazards analyses, death before PD diagnosis was treated as a censoring event. The association between IBS and subsequent PD risk was further evaluated using multivariable Cox proportional hazards regression models based on datasets constructed through exact matching on sex, age, and health screening year. The models adjusted for covariates available from health screening data, including sex, age, smoking status, alcohol consumption, BMI, blood pressure, fasting glucose, serum lipid levels, and income level, and additional subgroup analyses were performed. All statistical analyses were conducted using R software (version 4.4.1; R Foundation for Statistical Computing, Vienna, Austria), with statistical significance defined as a two-sided *p* value < 0.05.

### 2.7. Sensitivity Analysis

To assess the consistency of the association between IBS and PD, sensitivity analyses were conducted using alternative definitions of exposure and outcomes, including IBS subtypes and PD-related disorders. The corresponding ICD-10 code compositions are detailed in Supplementary Table S5.

## 3. Results

### 3.1. Incidence Rate Ratio Analysis

As shown in Table 2, a total of 142,668 patients with IBS and 285,336 matched reference individuals were included in the analysis. Over the entire follow-up period, the IBS cohort exhibited a significantly increased risk of developing PD compared with the reference cohort (IRR, 1.24; 95% CI, 1.14–1.36). However, subgroup analyses indicated that the magnitude of this association was not uniform across sex and age groups.

In age-stratified analyses, the risk of PD was higher in the IBS cohort than in the reference cohort among both individuals younger than 60 years (IRR, 1.32; 95% CI, 1.12–1.55) and those aged 60 years or older (IRR, 1.21; 95% CI, 1.09–1.35). Sex-stratified analyses likewise demonstrated significant risk increases in both men (IRR, 1.16; 95% CI, 1.02–1.32) and women (IRR, 1.32; 95% CI, 1.17–1.48). In subgroup analyses jointly stratified by age and sex, the IRR was highest among women younger than 60 years (IRR, 1.48; 95% CI, 1.19–1.83). Significant associations were also observed among men aged 60 years or older (IRR, 1.17; 95% CI, 1.00–1.36) and women aged 60 years or older (IRR, 1.25; 95% CI, 1.08–1.45), whereas the association was not statistically significant among men younger than 60 years (IRR, 1.14; 95% CI, 0.89–1.46).

In analyses stratified by smoking status, a significantly increased risk of PD was observed among current smokers (IRR, 1.45; 95% CI, 1.07–1.96) and former smokers (IRR, 1.24; 95% CI, 1.12–1.38) in the IBS cohort, whereas the association was not statistically significant among never smokers (IRR, 1.11; 95% CI, 0.90–1.36). Subgroup analyses according to alcohol consumption frequency, BMI, total cholesterol, and income level generally demonstrated a higher risk of PD among patients with IBS. Notably, significant associations were identified in individuals with BMI ≥ 25 kg/m^2^ (IRR, 1.25; 95% CI, 1.08–1.44), total cholesterol < 200 mg/dL (IRR, 1.33; 95% CI, 1.18–1.51), and those in the higher income group (IRR, 1.25; 95% CI, 1.11–1.40).

In the sensitivity analysis, the risk of PD among patients with IBS remained consistently and significantly elevated across all analyses applying alternative definitions of exposure and outcome conditions in the overall population. Detailed IRR estimates from these analyses are presented in Supplementary Table S6.

### 3.2. Cox Proportional Hazards Analysis

Cox proportional hazards regression analyses showed a significant overall association between IBS and incident PD, although this association was not uniformly observed across sex- and age-stratified subgroups after full adjustment (Supplementary Table S7). In the overall population, IBS was significantly associated with an increased risk of PD across all models, from the unadjusted model to the fully adjusted model (Model 3; HR, 1.38; 95% CI, 1.18–1.62). In the fully adjusted subgroup analyses, statistical significance was retained only among women (Model 3 HR, 1.53; 95% CI, 1.06–2.19), whereas the associations were not statistically significant among men or in either age-stratified subgroup. Sensitivity analyses further demonstrated that IBS remained significantly associated with an increased risk of PD in the fully adjusted model, including analyses using alternative exposure and outcome definitions (Supplementary Table S8). However, these overall findings should be interpreted together with the observed heterogeneity across sex- and age-stratified subgroups.

### 3.3. Kaplan–Meier Curve and Forest Plot Analysis

**Figure 3:**
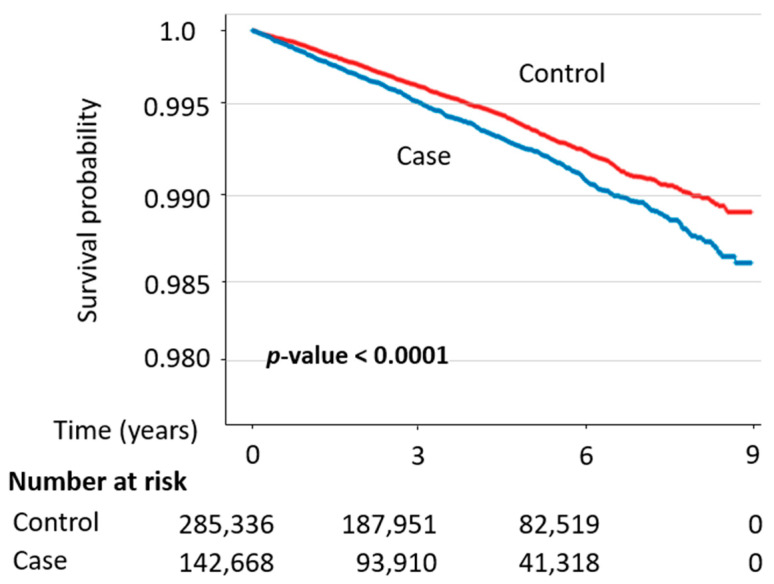
Kaplan–Meier curve for Parkinson’s disease-free survival in the IBS and matched reference cohorts.

**Figure 4:**
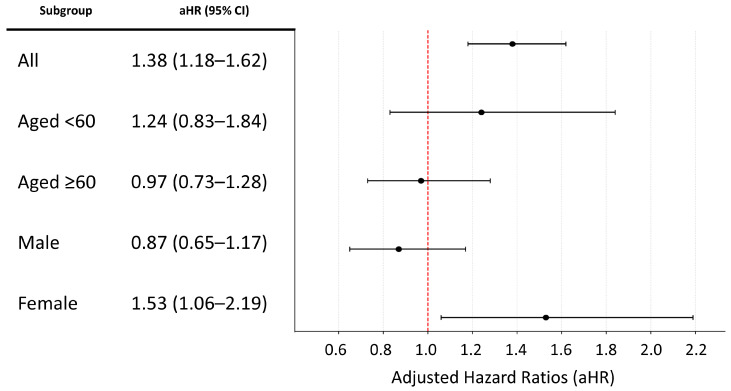
Forest plot of adjusted hazard ratios for the risk of Parkinson’s disease among individuals with irritable bowel syndrome. The red dashed line represents the hazard ratio reference value of 1.0.

Kaplan–Meier analyses showed significantly lower PD-free survival in the IBS cohort than in the matched reference cohort throughout the follow-up period compared with the reference cohort, and the difference between groups was statistically significant (log-rank *p* < 0.0001) (Figure 3). In forest plot analyses, a significant association between IBS and incident PD was observed in the overall population (adjusted HR [aHR], 1.38; 95% CI, 1.18–1.62), whereas subgroup analyses demonstrated a significant risk increase only among women (aHR, 1.53; 95% CI, 1.06–2.19); no statistically significant associations were observed among men or across age-stratified subgroups (Figure 4). Sensitivity analyses yielded concordant findings, with Kaplan–Meier curves similarly demonstrating significantly lower PD-free survival among patients with IBS, consistent with the main results (Supplementary Figure S1). In addition, forest plot-based sensitivity analyses consistently indicated higher risk estimates among women, with detailed results presented in Supplementary Figure S2.

## 4. Discussion

### 4.1. Overall Summary of Findings

This large-scale cohort study using NHIS data observed an association between IBS and an increased subsequent risk of PD. In IRR analyses, the IBS cohort showed a higher risk of PD than the reference cohort across the follow-up period; however, the magnitude and statistical significance of the association varied across sex- and age-stratified subgroups. The highest relative risk was observed among women younger than 60 years, while subgroup analyses according to smoking status, BMI, total cholesterol, and income level showed variable patterns of association. Sensitivity analyses using alternative exposure and outcome definitions yielded generally concordant results, supporting the robustness of the overall association. However, these findings should be interpreted cautiously given the observed subgroup heterogeneity and the observational claims-based design. Multivariable Cox regression analyses also supported an overall association, although statistical significance was not retained across all subgroups after full adjustment. The persistence of the association after full adjustment suggests that the increased PD risk among individuals with IBS may not be fully explained by measured lifestyle or metabolic factors. However, the observational design and heterogeneous subgroup findings preclude causal inference or uniform interpretation across all patient groups.

### 4.2. Previous Study

A Finnish cohort study [10] analyzed the risk of PD among 28,150 individuals with a primary diagnosis of IBS and 98,789 reference individuals between 1998 and 2014 without implementing a washout period. Although the IBS cohort showed an increased risk of PD over the entire follow-up period (aHR, 1.70; 95% CI, 1.27–2.26), this association was not statistically significant during longer-term follow-up beyond two years after the IBS diagnosis. In contrast, the present study applied a three-year washout period to minimize reverse causation and potential bias arising from the prodromal phase of PD and, through up to nine years of follow-up, demonstrated a significantly increased risk of PD following IBS (HR, 1.38; 95% CI, 1.18–1.62). Although the magnitude of the risk estimate observed in this study was lower than that reported in prior research, it may be interpreted as a more conservative effect estimate attributable to the more rigorous study design.

Whereas previous studies defined IBS based on a single ICD-10 diagnosis code [10,15], the present study applied a more stringent diagnostic criterion requiring at least two IBS diagnoses. In addition, prior research primarily focused on the association between a single IBS definition and PD, whereas this study further refined exposure definitions by stratifying IBS subtypes and extended outcome definitions to include PD-related disorders. By incorporating these sensitivity analyses, the present study evaluated the robustness and reproducibility of the observed associations.

### 4.3. Pathophysiological Mechanisms

IBS is a disorder characterized by dysfunction of the gut–brain axis and has been associated with low-grade mucosal immune activation, impaired intestinal barrier function, enteric nervous system inflammation, and gut microbiota dysbiosis [16,17]. These IBS-related alterations may be relevant to PD, as inflammatory processes involving microglial activation, cytokine-mediated signaling, blood–brain barrier dysfunction, and peripheral immune activation have been increasingly implicated in α-synuclein aggregation and dopaminergic neuronal vulnerability [18]. However, whether inflammation contributes to the initiation or progression of neurodegeneration, arises as a consequence of neuronal injury, or reflects a bidirectional process remains unresolved [19]. These mechanisms may provide biological plausibility for the observed IBS–PD association, but they do not establish a causal inflammatory pathway.

In IBS, persistent neural stimulation and low-grade inflammation within the enteric nervous system may create a local inflammatory environment that has been hypothesized to be relevant to α-synuclein-related pathological processes [20,21]. Evidence that pathologic α-synuclein formed in the gut can propagate to the central nervous system via the vagus nerve supports the “bottom-up hypothesis,” which posits that PD pathology may originate in the peripheral enteric nervous system [22]. In addition, impaired intestinal barrier function in IBS may increase gut permeability and facilitate the systemic translocation of inflammatory mediators, contributing to a state of low-grade systemic inflammation [23,24]. Such systemic inflammation could theoretically contribute to blood–brain barrier dysfunction and central neuroinflammatory responses, both of which have been implicated in PD pathophysiology [25].

Gut microbiota dysbiosis, which is commonly observed in patients with IBS, has also been linked to PD-related mechanisms [26,27]. Alterations in microbial composition may reduce neuroprotective metabolites and promote a proinflammatory milieu, which could be linked to α-synuclein aggregation and neuroinflammatory responses [28].

Metabolic and inflammatory dysregulation may provide an additional perspective on the IBS–PD association. Gut microbiota-derived metabolites in IBS have been linked to insulin resistance and impaired glucose control [29]. Glycemic variability and hypoglycemic episodes have also been discussed as clinically relevant factors in parkinsonian syndromes [30]. Although not specific to IBS, recent evidence suggesting that metabolic interventions such as SGLT-2 inhibitors may modulate neurodegenerative processes through vascular, mitochondrial, anti-inflammatory, and oxidative stress-related pathways supports the broader relevance of metabolic-inflammatory mechanisms in PD [31]. These observations do not establish a causal pathway specific to IBS-associated PD risk, but they suggest that metabolic and inflammatory dysregulation may warrant further investigation as a complementary mechanism.

### 4.4. Age

In crude IRR analyses, PD incidence was higher among patients with IBS in both age groups, younger than 60 years and 60 years or older, with a numerically higher relative estimate in the younger group. However, in fully adjusted Cox models, the association did not remain statistically significant in either age-stratified subgroup. Therefore, the age-stratified findings should not be interpreted as evidence of a uniform age-specific association. IRs were substantially higher among older adults, reflecting advanced age itself as a major determinant of PD occurrence [32,33]. Moreover, mortality may have influenced risk estimates among older adults because death before PD diagnosis was treated as a censoring event rather than as a competing event. Although the gut–brain axis hypothesis provides biological plausibility for further investigation, the present findings do not establish a consistent age-specific pattern of IBS-associated PD risk.

### 4.5. Sex

The association between IBS and incident PD was observed in both men and women; however, the magnitude of the relative risk was greater among women. In analyses jointly stratified by sex and age, statistically significant associations persisted only among women. These findings should be interpreted with caution and should not be considered definitive evidence of sex-specific differential effects.

Previous studies have suggested that women may exhibit greater susceptibility to alterations in bowel function, gut–immune interactions, autonomic nervous system responses, and hormonal influences [34,35]. The present study indicates sex-related heterogeneity in the association between IBS and PD, suggesting possible effect modification by sex. While the association persisted after multivariable adjustment in women, it was attenuated in men, potentially due to the greater contribution of age, lifestyle, and metabolic factors. These sex-stratified findings should therefore be interpreted cautiously as exploratory evidence rather than conclusive sex-specific differences.

### 4.6. Smoking Status

When evaluated using IRRs, a statistically significant increase in PD risk associated with IBS was observed among current and former smokers, whereas the association did not reach statistical significance among never smokers. However, comparisons based on IRs demonstrated consistently higher PD incidence among never smokers than among current or former smokers. This pattern is partially consistent with prior studies reporting an inverse association between cigarette smoking and PD risk [36,37]. These findings suggest that the relationship among smoking, IBS, and PD may be complex and may involve both inflammatory and non-inflammatory pathways. Nevertheless, the lack of statistical significance among never smokers may reflect differences in baseline risk, underscoring the need for more refined interaction analyses in future studies.

### 4.7. Alcohol

A significant association between IBS and incident PD was observed among non-drinkers, whereas no statistically significant associations were identified among individuals who consumed alcohol 1–2 times per week or three or more times per week. These findings are broadly consistent with prior studies reporting inverse or neutral associations between alcohol consumption and PD risk [38,39]. The absence of significant associations in higher-frequency drinking groups may be attributable to relatively smaller numbers of events, resulting in wider confidence intervals. This observation underscores the need for more granular analyses that account for heterogeneity in alcohol intake levels and drinking patterns.

### 4.8. Significance of the Study and Clinical Implications

The findings suggest that IBS may be associated with subsequent PD risk in certain patient subgroups and provide epidemiological support for further investigation of gut–brain axis-related mechanisms in PD [40,41]. However, because the association was not uniformly observed across sex- and age-stratified subgroups, these findings should be interpreted as exploratory and should not be regarded as evidence that IBS is an established clinical risk marker for PD.

This large population-based cohort study suggests that IBS may represent an epidemiological signal warranting further investigation in relation to neurodegenerative disease risk. The observed variation in relative and absolute risk patterns according to age, sex, smoking status, alcohol consumption, and metabolic factors highlights the need for more granular analyses incorporating medication use, healthcare utilization patterns, lifestyle factors, and detailed clinical phenotyping. Collectively, these findings support further research into gut–brain axis-related non-motor features and inflammatory, microbiome-related, intestinal barrier, and metabolic pathways, rather than immediate translation into routine neurological monitoring, screening, or preventive strategies.

### 4.9. Limitations

Although this study leveraged a large, nationwide cohort with extended follow-up, several limitations should be considered.

First, competing risk analyses were not performed, although death before PD diagnosis was treated as a censoring event in the Kaplan–Meier and Cox proportional hazards analyses. Because death may preclude subsequent PD diagnosis, particularly among older adults, this approach may have biased the estimated incidence and hazard of PD. Therefore, the findings, especially those from older age-stratified analyses, should be interpreted cautiously.

Second, although baseline characteristics were well balanced between the IBS and reference cohorts across all measured baseline covariates, unmeasured clinical heterogeneity in IBS phenotypes, comorbidities, medication exposure, healthcare-seeking patterns, lifestyle profiles, and socioeconomic backgrounds cannot be excluded, which may limit uniform interpretation of the observed IBS–PD association.

Third, residual confounding remains an important limitation because claims-based data lacked detailed information on medication use, healthcare-seeking behavior, and granular lifestyle factors such as diet, physical activity, stress, and temporal changes in these behaviors. Although key health screening variables were adjusted for, these unmeasured or insufficiently measured factors may have influenced the estimated association and limit causal interpretation.

Fourth, IBS and PD were defined using ICD-10 diagnostic codes in claims data, which may have introduced diagnostic misclassification. Although we applied a repeated-diagnosis criterion requiring at least two diagnoses to improve validity, claims-based coding may still have led to patient misclassification. Such misclassification may have reduced the reliability of the IBS case cohort by including individuals who did not fully meet clinical IBS criteria, potentially affecting the estimated magnitude of the IBS–PD association.

Fifth, detailed clinical information such as symptom severity, disease duration, treatment response, and prodromal or non-motor features of PD was unavailable. Therefore, we could not evaluate whether the IBS–PD association differed according to PD clinical subtype, disease course, or prodromal phenotype. More frequent healthcare encounters among patients with IBS may have increased PD detection and overestimated the association, although the three-year washout period was intended to reduce reverse causation and prodromal-detection bias.

### 4.10. Future Work

Future studies should validate the observed IBS–PD association using refined exposure and outcome definitions in independent datasets. In particular, studies incorporating detailed clinical phenotyping, including symptom severity, disease duration, treatment response, prodromal and non-motor features, and PD subtype information, are needed to determine whether the association differs according to clinical disease course.

In addition, studies integrating inflammatory biomarkers, microbiome profiles, and metabolic measures such as glycemic variability may help clarify the biological mechanisms underlying this association and identify potential targets for future therapeutic strategies. Replication in cohorts from other countries or independent administrative data-based populations would also be valuable for assessing the reproducibility and generalizability of the findings.

## 5. Conclusions

This large population-based cohort study using NHIS data observed an association between IBS and increased subsequent risk of PD. However, this association was not consistent across subgroups, with statistical significance in fully adjusted Cox models retained only among women. Given the observational claims-based design and the lack of detailed medication, lifestyle, clinical, and biological information, these findings should be interpreted as exploratory and hypothesis-generating. Further studies are warranted to validate the association and determine whether specific patient subgroups have clinically meaningful elevations in PD risk.

## Figures and Tables

**Table 1:** Baseline demographic characteristics of cohort including patients with IBS and matched reference cohort. BMI, body mass index; FBS, fasting blood sugar; IBS, irritable bowel syndrome.

		Case Group (*n* = 142,668) (%)	Control Group (*n* = 285,336) (%)	Standardized Difference
Age (years)	20–29	14,079 (9.87%)	28,158 (9.87%)	0.00
	30–39	25,818 (18.10%)	51,636 (18.10%)	
	40–49	35,368 (24.79%)	70,736 (24.79%)	
	50–59	37,908 (26.57%)	75,816 (26.57%)	
	60–69	21,170 (14.84%)	42,340 (14.84%)	
	≥70	8325 (5.84%)	16,650 (5.84%)	
Sex	Male	68,087 (47.72%)	136,174 (47.72%)	0.00
	Female	74,581 (52.28%)	149,162 (52.28%)	
Smoking status	Yes	24,584 (17.23%)	52,589 (18.43%)	0.05
	No	92,233 (64.65%)	185,224 (64.91%)	
	Ex-smoking	25,812 (18.09%)	47,338 (16.59%)	
Frequency of alcohol consumption (per week)	0	45,200 (31.68%)	92,696 (32.49%)	0.06
	1–2	50,128 (35.14%)	104,724 (36.70%)	
	≥3	14,292 (10.02%)	26,529 (9.30%)	
Weight (kg, mean ± SD)		64.52 ± 12.32	64.72 ± 12.38	0.02
Height (cm, mean ± SD)		163.15 ± 9.18	162.96 ± 9.24	0.02
BMI (kg/m^2^)	<18.5	4509 (3.16%)	8228 (2.88%)	0.04
	18.5 to <25	86,156 (60.39%)	168,644 (59.10%)	
	≥25	51,968 (36.43%)	108,384 (37.98%)	
Total cholesterol (mg/dL)	<200	50,362 (35.30%)	97,442 (34.15%)	0.07
	≥200	42,154 (29.55%)	80,258 (28.13%)	
Systolic Blood Pressure (mmHg)	<120	55,897 (39.18%)	110,262 (38.64%)	0.03
	120 to <140	68,608 (48.09%)	136,139 (47.71%)	
	≥140	17,464 (12.24%)	37,521 (13.15%)	
Diastolic Blood Pressure (mmHg)	<80	85,532 (59.95%)	169,552 (59.42%)	0.01
	80 to <90	43,961 (30.81%)	88,748 (31.10%)	
	≥90	12,476 (8.74%)	25,622 (8.98%)	
FBS (mg/dL)	<100	84,973 (59.56%)	162,963 (57.11%)	0.07
	100 to <126	44,856 (31.44%)	93,251 (32.68%)	
	≥126	12,133 (8.50%)	27,688 (9.70%)	
Income	Low	57,649 (40.41%)	114,224 (40.03%)	0.01
	High	80,354 (56.32%)	161,571 (56.62%)	

**Table 2:** Crude incidence rates and incidence rate ratios for Parkinson’s disease in the IBS and matched reference cohorts. IR, incidence rate; IRR, incidence rate ratio; IBS, irritable bowel syndrome.

		Case Cohort (*n* = 142,668)			Reference Cohort (*n* = 285,336)			IRR (95% CI)
		Cases	Person-Years	IR per 1000 Person-Years (95% CI)	Cases	Person-Years	IR per 1000 Person-Years (95% CI)	
All		801	612,304.26	1.31 (1.22–1.40)	1289	1,224,802.3	1.05 (1.00–1.11)	1.24 (1.14–1.36)
Age (years)	<60	246	475,031.93	0.52 (0.45–0.58)	373	950,036.88	0.39 (0.35–0.43)	1.32 (1.12–1.55)
	≥60	555	137,272.34	4.04 (3.71–4.39)	916	274,765.44	3.33 (3.12–3.55)	1.21 (1.09–1.35)
Sex	Male	356	290,155.93	1.23 (1.10–1.35)	613	580,113.35	1.06 (0.97–1.14)	1.16 (1.02–1.32)
	Female	445	322,148.34	1.38 (1.25–1.51)	676	644,688.97	1.05 (0.97–1.13)	1.32 (1.17–1.48)
Sex & Age (years)	Male, <60	100	228,200.9	0.44 (0.35–0.53)	175	456,201.68	0.38 (0.33–0.44)	1.14 (0.89–1.46)
	Male, ≥60	256	61,955.03	4.13 (3.63–4.65)	438	123,911.67	3.53 (3.20–3.87)	1.17 (1.00–1.36)
	Female, <60	146	246,831.03	0.59 (0.50–0.69)	198	493,835.19	0.40 (0.35–0.46)	1.48 (1.19–1.83)
	Female, ≥60	299	75,317.31	3.97 (3.53–4.42)	478	150,853.78	3.17 (2.89–3.45)	1.25 (1.08–1.45)
Smoking status	Yes	71	104,662.14	0.68 (0.53–0.84)	107	228,442.81	0.47 (0.38–0.56)	1.45 (1.07–1.96)
	Ex-Smoking	586	396,731.35	1.48 (1.36–1.60)	943	793,824.44	1.19 (1.11–1.26)	1.24 (1.12–1.38)
	No	144	110,742.04	1.30 (1.09–1.52)	237	201,809.55	1.17 (1.03–1.33)	1.11 (0.90–1.36)
Frequency of alcohol consumption (per week)	0	251	163,618.33	1.53 (1.34–1.73)	395	339,321.77	1.16 (1.05–1.28)	1.32 (1.12–1.54)
	1–2	120	175,840.97	0.68 (0.56–0.81)	210	370,106.74	0.57 (0.49–0.65)	1.20 (0.96–1.51)
	≥3	41	53,485.32	0.77 (0.54–1.01)	60	100,635.45	0.60 (0.45–0.76)	1.29 (0.86–1.91)
BMI (kg/m^2^)	<18.5	23	19,410.95	1.18 (0.72–1.70)	32	35,163.95	0.91 (0.60–1.25)	1.30 (0.76–2.22)
	18.5 to <25	467	375,621.75	1.24 (1.13–1.36)	733	733,699.35	1.00 (0.93–1.07)	1.24 (1.11–1.40)
	≥25	311	217,102.04	1.43 (1.28–1.59)	523	455,629.21	1.15 (1.05–1.25)	1.25 (1.08–1.44)
Total cholesterol (mg/dL)	<200	423	261,712.47	1.62 (1.46–1.77)	618	509,400.1	1.21 (1.12–1.31)	1.33 (1.18–1.51)
	≥200	226	214,345.32	1.05 (0.92–1.19)	393	414,291.45	0.95 (0.86–1.04)	1.11 (0.94–1.31)
Income	Low	308	244,969.64	1.26 (1.12–1.40)	501	488,759.27	1.03 (0.94–1.12)	1.23 (1.06–1.41)
	High	467	347,368	1.34 (1.22–1.47)	749	694,575.87	1.08 (1.00–1.16)	1.25 (1.11–1.40)

## Data Availability

The data that support the findings of this study are available from the corresponding author upon reasonable request. The data are not publicly available due to restrictions related to privacy and ethical considerations, as they are derived from the Korean National Health Insurance Service (NHIS) database.

## Supplementary Materials

The following supporting information can be downloaded at https://www.mdpi.com/article/10.3390/healthcare14101329/s1, Figure S1: Kaplan–Meier curve for outcome-free survival following IBS (irritable bowel syndrome) diagnosis; Figure S2: Forest plot of adjusted hazard ratios (aHRs) with 95% confidence intervals for the risk of outcome among individuals with IBS (irritable bowel syndrome), stratified by sex and age; Table S1: ICD-10 codes used to define IBS (irritable bowel syndrome) exposure and Parkinson’s disease outcome; Table S2: Mean follow-up duration in IBS (irritable bowel syndrome) cases and matched controls; Table S3: ICD-10 codes used for exclusion criteria in study cohort selection; Table S4: Baseline demographic characteristics of patients with IBS (irritable bowel syndrome) and matched control group. BMI, body mass index; FBS, fasting blood sugar; Table S5: ICD-10 codes used to define the exposure IBS (irritable bowel syndrome) and the outcomes; Table S6: Crude incidence rates and incidence rate ratios of Parkinson’s disease among IBS (irritable bowel syndrome) cases and matched controls; Table S7: Cox proportional hazards analysis for the association between IBS (irritable bowel syndrome) and risk of Parkinson’s disease; Table S8: Cox proportional hazards analysis of the association between IBS (irritable bowel syndrome), with or without diarrhea, and the risk of Parkinson’s disease.

## Abbreviations

The following abbreviations are used in this manuscript:

IBS	Irritable bowel syndrome
PD	Parkinson’s disease
NHIS	National Health Insurance Service
ICD-10	International Classification of Diseases, 10th Revision
IR	Incidence rate
IRR	Incidence rate ratio
HR	Hazard ratio
aHR	Adjusted hazard ratio
CI	Confidence interval
BMI	Body mass index
