# Supplementary material for: Long-Term Risk of Parkinson’s Disease Following Irritable Bowel Syndrome: A Nationwide Population-Based Cohort Study
Source: Healthcare (Basel). 2026 May 13;14(10):1329. doi: 10.3390/healthcare14101329 (PMC13205689; doi:10.3390/healthcare14101329)

Supplementary Materials

Table S1. ICD-10 codes used to define IBS (irritable bowel syndrome) exposure and Parkinson’s disease outcome.

**Disease**	**ICD-10 code**
Exposure	
Irritable bowel syndrome	K58
Outcome	
Parkinson’s disease, idiopathic	G20

Table S2. Mean follow-up duration in IBS (irritable bowel syndrome) cases and matched controls

Exposure disease	Group	Events, n	Follow-up, years (mean ± SD)
IBS	Control	1289	4.29 ± 2.49
	Case	801	4.29 ± 2.49
IBS with diarrhea	Control	666	4.55 ± 2.41
	Case	376	4.53 ± 2.42
IBS without diarrhea	Control	996	4.10 ± 2.51
	Case	669	4.10 ± 2.51

Table S3. ICD-10 codes used for exclusion criteria in study cohort selection

IBS: irritable bowel syndrome

Disease	ICD-10 code	Notes
**Parkinson's disease and related parkinsonian disorders**		
Parkinson’s disease, idiopathic	G20	Primary outcome
Secondary parkinsonism	G21	Drug-induced or vascular parkinsonism
Parkinsonism in other diseases	G22	Parkinsonism associated with other medical conditions
Other degenerative diseases of basal ganglia	G23	Atypical parkinsonian syndromes
**Major neurodegenerative disorders**		
Alzheimer’s disease	G30	
Vascular dementia	F01	
Unspecified dementia	F03	
Dementia with Lewy bodies	G31.8	
Other degenerative diseases of the nervous system	G31	
**Structural disorders of the central nervous system**		
Ischemic stroke	I63	
Hemorrhagic stroke	I60, I61, I62	
Sequelae of cerebrovascular disease	I69	
Traumatic brain injury	S06	
Malignant neoplasm of brain	C71	
Benign neoplasm of brain	D33	
Neoplasm of uncertain or unknown behavior of brain	D43	
**Organic gastrointestinal diseases that may compromise the validity of IBS diagnosis**		
Crohn’s disease	K50	
Ulcerative colitis	K51	
Other inflammatory bowel diseases	K52	
Ischemic colitis	K55	
Celiac disease	K90.0	
Intestinal malabsorption	K90	
Colorectal cancer	C18, C19, C20	
**Systemic diseases strongly confounding the risk of parkinson’s disease**		
Systemic lupus erythematosus	M32	
Rheumatoid arthritis	M05, M06	
Vasculitis	M30, M31	
Sjögren syndrome	M35.0	
Chronic kidney disease (stage ≥4)	N18.4, N18.5, N18.6	
Chronic liver failure / hepatic encephalopathy	K72	

Table S4. Baseline demographic characteristics of patients with IBS (irritable bowel syndrome) and matched control group. BMI, body mass index; FBS, fasting blood sugar
1. IBS

		Case group (n = 142,302) (%)	Control group (n = 284,604) (%)	Standardized difference
Age (years)	20–29	14064 (9.88%)	28128 (9.88%)	0.00
	30–39	25788 (18.12%)	51576 (18.12%)	
	40–49	35299 (24.81%)	70598 (24.81%)	
	50–59	37830 (26.58%)	75660 (26.58%)	
	60–69	21068 (14.81%)	42136 (14.81%)	
	≥70	8253 (5.80%)	16506 (5.80%)	
Sex	Male	67934 (47.74%)	135868 (47.74%)	0.00
	Female	74368 (52.26%)	148736 (52.26%)	
Smoking status	Yes	24534 (17.24%)	52639 (18.50%)	0.05
	No	91972 (64.63%)	184177 (64.71%)	
	Ex-smoking	25757 (18.10%)	47623 (16.73%)	
Frequency of alcohol consumption (per week)	0	45042 (31.65%)	92238 (32.41%)	0.06
	1–2	50075 (35.19%)	104822 (36.83%)	
	≥3	14274 (10.03%)	26551 (9.33%)	
Weight (kg, mean ± SD)		64.52 ± 12.32	64.74 ± 12.36	0.02
Height (cm, mean ± SD)		163.16 ± 9.17	162.97 ± 9.22	0.02
BMI (kg/m^2)	<18.5	4494 (3.16%)	8044 (2.83%)	0.05
	18.5 to <25	85964 (60.41%)	168176 (59.09%)	
	≥25	51810 (36.41%)	108297 (38.05%)	
Total cholesterol (mg/dL)	<200	50198 (35.28%)	97137 (34.13%)	0.07
	≥200	42065 (29.56%)	79991 (28.11%)	
Systolic Blood Pressure (mmHg)	<120	55771 (39.19%)	110125 (38.69%)	0.03
	120 to <140	68441 (48.10%)	135991 (47.78%)	
	≥140	17397 (12.23%)	37192 (13.07%)	
Diastolic Blood Pressure (mmHg)	<80	85310 (59.95%)	169172 (59.44%)	0.02
	80 to <90	43850 (30.81%)	88669 (31.16%)	
	≥90	12449 (8.75%)	25468 (8.95%)	
FBS (mg/dL)	<100	84792 (59.59%)	162715 (57.17%)	0.07
	100 to <126	44731 (31.43%)	92757 (32.59%)	
	≥126	12078 (8.49%)	27816 (9.77%)	
Income	Low	57476 (40.39%)	113572 (39.91%)	0.01
	High	80171 (56.34%)	161570 (56.77%)	

2. IBS with diarrhea

(A) Outcome - Parkinson's disease, idiopathic				
		**Case group (n = 55,739) (%)**	**Control group (n = 111,478) (%)**	**Standardized difference**
Age (years)	20–29	6161 (11.05%)	12322 (11.05%)	0.00
	30–39	8870 (15.91%)	17740 (15.91%)	
	40–49	11714 (21.02%)	23428 (21.02%)	
	50–59	14394 (25.82%)	28788 (25.82%)	
	60–69	9912 (17.78%)	19824 (17.78%)	
	≥70	4688 (8.41%)	9376 (8.41%)	
Sex	Male	27236 (48.86%)	54472 (48.86%)	0.00
	Female	28503 (51.14%)	57006 (51.14%)	
Smoking status	Yes	9062 (16.26%)	19791 (17.75%)	0.06
	No	36095 (64.76%)	72473 (65.01%)	
	Ex-smoking	10570 (18.96%)	19146 (17.17%)	
Frequency of alcohol consumption (per week)	0	17838 (32.00%)	35981 (32.28%)	0.07
	1–2	18014 (32.32%)	38570 (34.60%)	
	≥3	5115 (9.18%)	10061 (9.03%)	
Weight (kg, mean ± SD)		61.18 ± 11.08	64.14 ± 12.30	0.04
Height (cm, mean ± SD)		158.67 ± 9.20	162.98 ± 9.34	0.02
BMI (kg/m^2)	<18.5	1950 (3.50%)	3173 (2.85%)	0.07
	18.5 to <25	33974 (60.95%)	66136 (59.33%)	
	≥25	19799 (35.52%)	42133 (37.79%)	
Total cholesterol (mg/dL)	<200	21507 (38.59%)	40727 (36.53%)	0.07
	≥200	16150 (28.97%)	31479 (28.24%)	
Systolic Blood Pressure (mmHg)	<120	21551 (38.66%)	42096 (37.76%)	0.04
	120 to <140	26880 (48.22%)	53588 (48.07%)	
	≥140	6932 (12.44%)	15208 (13.64%)	
Diastolic Blood Pressure (mmHg)	<80	33551 (60.19%)	66383 (59.55%)	0.03
	80 to <90	17208 (30.87%)	34593 (31.03%)	
	≥90	4604 (8.26%)	9917 (8.90%)	
FBS (mg/dL)	<100	33072 (59.33%)	63773 (57.21%)	0.06
	100 to <126	17337 (31.10%)	36465 (32.71%)	
	≥126	4947 (8.88%)	10647 (9.55%)	
Income	Low	22263 (39.94%)	43699 (39.20%)	0.02
	High	31737 (56.94%)	63953 (57.37%)	

	High	63714 (56.46%)	128247 (56.82%)	
**(B) Outcome - Overall Parkinson's disease related disorders**				
		**Case group (n = 55,565) (%)**	**Control group (n = 111,130) (%)**	**Standardized difference**
Age (years)	20–29	6150 (11.07%)	12300 (11.07%)	0.00
	30–39	8855 (15.94%)	17710 (15.94%)	
	40–49	11686 (21.03%)	23372 (21.03%)	
	50–59	14355 (25.83%)	28710 (25.83%)	
	60–69	9871 (17.76%)	19742 (17.76%)	
	≥70	4648 (8.36%)	9296 (8.36%)	
Sex	Male	27165 (48.89%)	54330 (48.89%)	0.00
	Female	28400 (51.11%)	56800 (51.11%)	
Smoking status	Yes	9033 (16.26%)	19588 (17.63%)	0.06
	No	35973 (64.74%)	72130 (64.91%)	
	Ex-smoking	10547 (18.98%)	19352 (17.41%)	
Frequency of alcohol consumption (per week)	0	17767 (31.98%)	36152 (32.53%)	0.08
	1–2	17980 (32.36%)	38291 (34.46%)	
	≥3	5110 (9.20%)	10122 (9.11%)	
Weight (kg, mean ± SD)		64.14 ± 12.30	64.54 ± 12.34	0.03
Height (cm, mean ± SD)		162.99 ± 9.34	162.84 ± 9.33	0.02
BMI (kg/m^2)	<18.5	1949 (3.51%)	3210 (2.89%)	0.07
	18.5 to <25	33885 (60.98%)	66004 (59.39%)	
	≥25	19715 (35.48%)	41877 (37.68%)	
Total cholesterol (mg/dL)	<200	21438 (38.58%)	40654 (36.58%)	0.07
	≥200	16106 (28.99%)	31297 (28.16%)	
Systolic Blood Pressure (mmHg)	<120	21489 (38.67%)	41978 (37.77%)	0.04
	120 to <140	26804 (48.24%)	53462 (48.11%)	
	≥140	6899 (12.42%)	15079 (13.57%)	
Diastolic Blood Pressure (mmHg)	<80	33455 (60.21%)	66228 (59.60%)	0.03
	80 to <90	17150 (30.86%)	34511 (31.05%)	
	≥90	4587 (8.26%)	9780 (8.80%)	
FBS (mg/dL)	<100	32989 (59.37%)	63911 (57.51%)	0.06
	100 to <126	17274 (31.09%)	35955 (32.35%)	
	≥126	4922 (8.86%)	10648 (9.58%)	
Income	Low	22176 (39.91%)	43874 (39.48%)	0.02
	High	31655 (56.97%)	63417 (57.07%)	

3. IBS without diarrhea

(A) Outcome - Parkinson's disease, idiopathic				
		**Case group (n = 112,852) (%)**	**Control group (n = 225,704) (%)**	**Standardized difference**
Age (years)	20–29	9105 (8.07%)	18210 (8.07%)	0.00
	30–39	19669 (17.43%)	39338 (17.43%)	
	40–49	28502 (25.26%)	57004 (25.26%)	
	50–59	31558 (27.96%)	63116 (27.96%)	
	60–69	17538 (15.54%)	35076 (15.54%)	
	≥70	6480 (5.74%)	12960 (5.74%)	
Sex	Male	52445 (46.47%)	104890 (46.47%)	0.00
	Female	60407 (53.53%)	120814 (53.53%)	
Smoking status	Yes	18406 (16.31%)	39280 (17.40%)	0.05
	No	74081 (65.64%)	148587 (65.83%)	
	Ex-smoking	20328 (18.01%)	37703 (16.70%)	
Frequency of alcohol consumption (per week)	0	37404 (33.14%)	76227 (33.77%)	0.06
	1–2	39781 (35.25%)	83115 (36.82%)	
	≥3	11232 (9.95%)	21167 (9.38%)	
Weight (kg, mean ± SD)		64.18 ± 12.13	64.50 ± 12.19	0.03
Height (cm, mean ± SD)		162.82 ± 9.09	162.66 ± 9.18	0.02
BMI (kg/m^2)	<18.5	3467 (3.07%)	6027 (2.67%)	0.06
	18.5 to <25	68729 (60.90%)	134093 (59.41%)	
	≥25	40632 (36.00%)	85529 (37.89%)	
Total cholesterol (mg/dL)	<200	38234 (33.88%)	75516 (33.46%)	0.06
	≥200	33121 (29.35%)	62047 (27.49%)	
Systolic Blood Pressure (mmHg)	<120	43825 (38.83%)	86245 (38.21%)	0.03
	120 to <140	54286 (48.10%)	108241 (47.96%)	
	≥140	14097 (12.49%)	30010 (13.30%)	
Diastolic Blood Pressure (mmHg)	<80	67652 (59.95%)	134487 (59.59%)	0.01
	80 to <90	34631 (30.69%)	69734 (30.90%)	
	≥90	9925 (8.79%)	20276 (8.98%)	
FBS (mg/dL)	<100	66615 (59.03%)	127952 (56.69%)	0.07
	100 to <126	35971 (31.87%)	74378 (32.95%)	
	≥126	9617 (8.52%)	22148 (9.81%)	
Income	Low	45552 (40.36%)	89968 (39.86%)	0.01
	High	63714 (56.46%)	128247 (56.82%)	

(B) Outcome - Overall Parkinson’s disease related disorders				
		**Case group (n = 112,554) (%)**	**Control group (n = 225,108) (%)**	**Standardized difference**
Age (years)	20–29	9099 (8.08%)	18198 (8.08%)	0.00
	30–39	19643 (17.45%)	39286 (17.45%)	
	40–49	28448 (25.27%)	56896 (25.27%)	
	50–59	31486 (27.97%)	62972 (27.97%)	
	60–69	17459 (15.51%)	34918 (15.51%)	
	≥70	6419 (5.70%)	12838 (5.70%)	
Sex	Male	52321 (46.49%)	104642 (46.49%)	0.00
	Female	60233 (53.51%)	120466 (53.51%)	
Smoking status	Yes	18366 (16.32%)	39040 (17.34%)	0.05
	No	73864 (65.63%)	148307 (65.88%)	
	Ex-smoking	20287 (18.02%)	37617 (16.71%)	
Frequency of alcohol consumption (per week)	0	37276 (33.12%)	75850 (33.69%)	0.05
	1–2	39729 (35.30%)	82731 (36.75%)	
	≥3	11218 (9.97%)	21261 (9.44%)	
Weight (kg, mean ± SD)		64.19 ± 12.13	64.49 ± 12.22	0.02
Height (cm, mean ± SD)		162.83 ± 9.09	162.66 ± 9.15	0.02
BMI (kg/m^2)	<18.5	3452 (3.07%)	6412 (2.85%)	0.06
	18.5 to <25	68569 (60.92%)	132848 (59.02%)	
	≥25	40510 (35.99%)	85779 (38.11%)	
Total cholesterol (mg/dL)	<200	38103 (33.85%)	74954 (33.30%)	0.06
	≥200	33046 (29.36%)	62010 (27.55%)	
Systolic Blood Pressure (mmHg)	<120	43719 (38.84%)	86031 (38.22%)	0.03
	120 to <140	54149 (48.11%)	108095 (48.02%)	
	≥140	14046 (12.48%)	29790 (13.23%)	
Diastolic Blood Pressure (mmHg)	<80	67468 (59.94%)	134424 (59.72%)	0.01
	80 to <90	34546 (30.69%)	69630 (30.93%)	
	≥90	9900 (8.80%)	19862 (8.82%)	
FBS (mg/dL)	<100	66468 (59.05%)	127349 (56.57%)	0.07
	100 to <126	35871 (31.87%)	74203 (32.96%)	
	≥126	9569 (8.50%)	22352 (9.93%)	
Income	Low	45410 (40.35%)	89677 (39.84%)	0.01
	High	63566 (56.48%)	128124 (56.92%)	

Table S5. ICD-10 codes used to define the exposure IBS (irritable bowel syndrome) and the outcomes.

Disease	ICD-10 code
**Sensitivity analysis 1**	
Exposure	
IBS	K58
Outcome	
Overall Parkinson's disease related disorders	G20, G21, G22, G23
**Sensitivity analysis 2**	
Exposure	
IBS with diarrhea	K58.0, K58.1
IBS without diarrhea	K58.2, K58.3, K58.8, K58.9
Outcome	
Parkinson's disease, idiopathic	G20
Overall Parkinson's disease related disorders	G20, G21, G22, G23

Table S6. Crude incidence rates and incidence rate ratios of Parkinson's disease among IBS (irritable bowel syndrome) cases and matched controls.

IR, incidence rate; IRR, incidence rate ratio.

1. IBS

		**Case cohort (n =142,302)**			**Reference cohort (n = 284,604)**			**IRR (95% CI)**
		**Cases**	**Person-years**	**IR per 1000 person-years (95% CI)**	**Cases**	**Person-years**	**IR per 1000 person-years (95% CI)**	
All		1160	609815.64	1.90 (1.79–2.01)	1745	1220738.1	1.43 (1.36–1.50)	1.33 (1.24–1.43)
Age (years)	<60	407	473798.68	0.86 (0.78–0.94)	626	947681.24	0.66 (0.61–0.71)	1.30 (1.15–1.47)
	≥60	753	136016.96	5.54 (5.15–5.93)	1119	273056.89	4.10 (3.86–4.34)	1.35 (1.23–1.48)
Sex	Male	512	289108.35	1.77 (1.62–1.93)	763	578644.08	1.32 (1.23–1.41)	1.34 (1.20–1.50)
	Female	648	320707.3	2.02 (1.87–2.18)	982	642094.05	1.53 (1.43–1.63)	1.32 (1.20–1.46)
Sex & Age group60 (years)	Male, <60	162	227660.79	0.71 (0.61–0.82)	258	455310.64	0.57 (0.50–0.64)	1.26 (1.03–1.53)
	Male, ≥60	350	61447.55	5.70 (5.11–6.30)	505	123333.44	4.09 (3.74–4.46)	1.39 (1.21–1.59)
	Female, <60	245	246137.89	1.00 (0.87–1.12)	368	492370.6	0.75 (0.67–0.82)	1.33 (1.13–1.57)
	Female, ≥60	403	74569.4	5.40 (4.88–5.94)	614	149723.45	4.10 (3.78–4.43)	1.32 (1.16–1.49)
Smoking status	Yes	120	104336.94	1.15 (0.95–1.36)	186	228219.78	0.82 (0.70–0.93)	1.41 (1.12–1.78)
	Ex-Smoking	836	394964.04	2.12 (1.97–2.26)	1291	789364.58	1.64 (1.55–1.73)	1.29 (1.19–1.41)
	No	204	110345.93	1.85 (1.59–2.10)	268	202494.46	1.32 (1.17–1.49)	1.40 (1.16–1.68)
Frequency of alcohol	0	354	162962.1	2.17 (1.95–2.40)	492	336848.55	1.46 (1.33–1.59)	1.49 (1.30–1.70)

consumption (per week)	1–2	183	175518.25	1.04 (0.89–1.20)	247	371344.92	0.67 (0.58–0.75)	1.57 (1.29–1.90)
	≥3	66	53359.73	1.24 (0.96–1.54)	107	100410.19	1.07 (0.87–1.27)	1.16 (0.85–1.58)
BMI (kg/m^2)	<18.5	34	19334.6	1.76 (1.19–2.38)	59	34297.73	1.72 (1.28–2.19)	1.02 (0.67–1.56)
	18.5 to <25	679	374279.1	1.81 (1.68–1.95)	1009	731188.8	1.38 (1.30–1.47)	1.31 (1.19–1.45)
	≥25	447	216039.32	2.07 (1.88–2.26)	677	454840.19	1.49 (1.38–1.60)	1.39 (1.23–1.57)
Total cholesterol (mg/dL)	<200	600	260451.96	2.30 (2.12–2.49)	850	507462.84	1.67 (1.56–1.79)	1.38 (1.24–1.53)
	≥200	346	213549.23	1.62 (1.45–1.79)	554	412398.58	1.34 (1.23–1.46)	1.21 (1.05–1.38)
Income	Low	452	243836.2	1.85 (1.69–2.03)	708	485979.78	1.46 (1.35–1.57)	1.27 (1.13–1.43)
	High	671	346080.08	1.94 (1.79–2.09)	984	693521.8	1.42 (1.33–1.51)	1.37 (1.24–1.51)

2. IBS with diarrhea

**(A) Outcome - Parkinson's disease, idiopathic**								
		**Case cohort (n =55,739)**			**Reference cohort (n = 111,478)**			**IRR (95% CI)**
		**Cases**	**Person-years**	**IR per 1000 person-years (95% CI)**	**Cases**	**Person-years**	**IR per 1000 person-years (95% CI)**	
All		376	252430.53	1.49 (1.34–1.64)	666	506992.81	1.31 (1.22–1.41)	1.13 (1.00–1.29)
Age (years)	<60	98	183563.52	0.53 (0.43–0.64)	165	367790.97	0.45 (0.38–0.52)	1.19 (0.93–1.53)
	≥60	278	68867.02	4.04 (3.57–4.52)	501	139201.84	3.60 (3.29–3.92)	1.12 (0.97–1.30)
Sex	Male	171	122801.62	1.39 (1.19–1.60)	317	246756.5	1.28 (1.15–1.43)	1.08 (0.90–1.31)

	Female	205	129628.91	1.58 (1.37–1.81)	349	260236.31	1.34 (1.20–1.48)	1.18 (0.99–1.40)
Sex & Age group60 (years)	Male, <60	38	91258.4	0.42 (0.28–0.56)	84	182828.74	0.46 (0.37–0.56)	0.91 (0.62–1.33)
	Male, ≥60	133	31543.23	4.22 (3.52–4.95)	233	63927.76	3.64 (3.19–4.11)	1.16 (0.93–1.43)
	Female, <60	60	92305.12	0.65 (0.49–0.82)	81	184962.24	0.44 (0.35–0.54)	1.48 (1.06–2.07)
	Female, ≥60	145	37323.79	3.88 (3.27–4.53)	268	75274.07	3.56 (3.14–4.00)	1.09 (0.89–1.34)
Smoking status	Yes	32	41224.82	0.78 (0.51–1.07)	55	91823.05	0.60 (0.45–0.76)	1.30 (0.84–2.00)
	Ex-Smoking	273	163251.58	1.67 (1.48–1.87)	502	328282.19	1.53 (1.40–1.66)	1.09 (0.94–1.27)
	No	71	47902.99	1.48 (1.15–1.84)	109	86595.13	1.26 (1.03–1.50)	1.18 (0.87–1.59)
Frequency of alcohol consumption (per week)	0	119	67256.1	1.77 (1.46–2.10)	203	138629.06	1.46 (1.27–1.67)	1.21 (0.96–1.52)
	1–2	47	67709.51	0.69 (0.50–0.90)	89	147382.93	0.60 (0.48–0.73)	1.15 (0.81–1.64)
	≥3	26	20773.97	1.25 (0.82–1.73)	44	40702.71	1.08 (0.76–1.40)	1.16 (0.71–1.88)
BMI (kg/m^2)	<18.5	12	8985.68	1.34 (0.67–2.11)	18	14357.67	1.25 (0.70–1.88)	1.07 (0.51–2.21)
	18.5 to <25	214	156040.18	1.37 (1.19–1.56)	398	304980.87	1.30 (1.18–1.44)	1.05 (0.89–1.24)
	≥25	150	87327.05	1.72 (1.44–1.99)	249	187489.82	1.33 (1.17–1.49)	1.29 (1.06–1.58)
Total cholesterol (mg/dL)	<200	193	114931.83	1.68 (1.44–1.92)	357	220321.28	1.62 (1.45–1.79)	1.04 (0.87–1.23)
	≥200	107	85910.95	1.25 (1.01–1.49)	198	170381.47	1.16 (1.00–1.33)	1.07 (0.85–1.36)
Income	Low	134	100187.54	1.34 (1.12–1.57)	219	198305.53	1.10 (0.96–1.25)	1.21 (0.98–1.50)
	High	228	144271.08	1.58 (1.38–1.79)	423	291201.01	1.45 (1.32–1.59)	1.09 (0.93–1.28)

(B) Outcome - Overall Parkinson's disease related disorders								
		Case cohort (n =55,565)			Reference cohort (n = 111,130)			IRR (95% CI)
		Cases	Person-years	IR per 1000 person-years (95% CI)	Cases	Person-years	IR per 1000 person-years (95% CI)	
All		576	251138.95	2.29 (2.11–2.48)	882	504796.2	1.75 (1.63–1.86)	1.31 (1.18–1.46)
Age (years)	<60	186	182933.03	1.02 (0.87–1.16)	272	366831.91	0.74 (0.65–0.83)	1.37 (1.14–1.65)
	≥60	390	68205.91	5.72 (5.16–6.29)	610	137964.29	4.42 (4.07–4.78)	1.29 (1.14–1.47)
Sex	Male	254	122279.01	2.08 (1.82–2.34)	431	245865.28	1.75 (1.59–1.92)	1.18 (1.01–1.38)
	Female	322	128859.94	2.50 (2.23–2.78)	451	258930.92	1.74 (1.58–1.90)	1.43 (1.24–1.66)
Sex & Age group60 (years)	Male, <60	72	90980.74	0.79 (0.62–0.98)	125	182471.82	0.69 (0.57–0.81)	1.16 (0.86–1.54)
	Male, ≥60	182	31298.27	5.82 (4.98–6.68)	306	63393.46	4.83 (4.29–5.38)	1.20 (1.00–1.45)
	Female, <60	114	91952.29	1.24 (1.02–1.47)	147	184360.09	0.80 (0.67–0.93)	1.55 (1.22–1.99)
	Female, ≥60	208	36907.65	5.64 (4.88–6.42)	304	74570.83	4.08 (3.62–4.55)	1.38 (1.16–1.65)
Smoking status	Yes	61	41051.48	1.49 (1.12–1.88)	88	90589.97	0.97 (0.77–1.18)	1.53 (1.10–2.12)
	Ex-Smoking	409	162348.99	2.52 (2.28–2.77)	634	326633.73	1.94 (1.79–2.09)	1.30 (1.15–1.47)
	No	106	47687.32	2.22 (1.80–2.66)	159	87313.87	1.82 (1.55–2.11)	1.22 (0.95–1.56)
Frequency of alcohol consumption (per week)	0	178	66905.12	2.66 (2.27–3.06)	258	139118.26	1.85 (1.63–2.08)	1.43 (1.19–1.74)
	1–2	89	67515.01	1.32 (1.05–1.60)	138	146631.14	0.94 (0.78–1.10)	1.40 (1.07–1.83)

	≥3	33	20728.59	1.59 (1.06–2.17)	59	41009.24	1.44 (1.07–1.83)	1.11 (0.72–1.69)
BMI (kg/m^2)	<18.5	17	8964.31	1.90 (1.00–2.90)	17	14684.46	1.16 (0.61–1.77)	1.64 (0.84–3.21)
	18.5 to <25	341	155344.73	2.20 (1.96–2.43)	534	303746.3	1.76 (1.61–1.91)	1.25 (1.09–1.43)
	≥25	218	86752.29	2.51 (2.19–2.85)	330	186169.08	1.77 (1.58–1.97)	1.42 (1.19–1.68)
Total cholesterol (mg/dL)	<200	290	114322.15	2.54 (2.25–2.83)	472	220007.42	2.15 (1.95–2.34)	1.18 (1.02–1.37)
	≥200	174	85464.77	2.04 (1.74–2.34)	251	168596.6	1.49 (1.30–1.68)	1.37 (1.13–1.66)
Income	Low	221	99561.93	2.22 (1.93–2.52)	335	198032.06	1.69 (1.51–1.87)	1.31 (1.11–1.56)
	High	336	143648.93	2.34 (2.10–2.59)	516	289247.51	1.78 (1.63–1.94)	1.31 (1.14–1.50)

3. IBS without diarrhea

**(A) Outcome - Parkinson's disease, idiopathic**								
		**Case cohort (n =112,852)**			**Reference cohort (n = 225,704)**			**IRR (95% CI)**
		**Cases**	**Person-years**	**IR per 1000 person-years (95% CI)**	**Cases**	**Person-years**	**IR per 1000 person-years (95% CI)**	
All		669	462169.62	1.45 (1.34–1.56)	996	924750.94	1.08 (1.01–1.14)	1.34 (1.22–1.48)
Age (years)	<60	206	354945.27	0.58 (0.50–0.66)	285	709833.84	0.40 (0.36–0.45)	1.45 (1.21–1.73)
	≥60	463	107224.34	4.32 (3.93–4.72)	711	214917.1	3.31 (3.07–3.55)	1.31 (1.16–1.47)
Sex	Male	308	212396.16	1.45 (1.29–1.61)	447	424934.26	1.05 (0.96–1.15)	1.38 (1.19–1.59)
	Female	361	249773.45	1.45 (1.30–1.60)	549	499816.68	1.10 (1.01–1.19)	1.32 (1.15–1.50)

Sex & Age group60 (years)	Male, <60	84	164776.83	0.51 (0.41–0.62)	115	329439.12	0.35 (0.29–0.41)	1.46 (1.10–1.93)
	Male, ≥60	224	47619.34	4.70 (4.09–5.33)	332	95495.15	3.48 (3.11–3.85)	1.35 (1.14–1.60)
	Female, <60	122	190168.45	0.64 (0.53–0.76)	170	380394.73	0.45 (0.38–0.52)	1.44 (1.14–1.81)
	Female, ≥60	239	59605	4.01 (3.51–4.53)	379	119421.95	3.17 (2.86–3.50)	1.26 (1.07–1.49)
Smoking status	Yes	61	74404.44	0.82 (0.62–1.03)	77	162248.31	0.47 (0.37–0.59)	1.73 (1.23–2.42)
	Ex-Smoking	495	304613.33	1.63 (1.48–1.77)	740	608609.48	1.22 (1.13–1.30)	1.34 (1.19–1.50)
	No	113	82996.87	1.36 (1.12–1.61)	179	153387.89	1.17 (1.00–1.34)	1.17 (0.92–1.48)
Frequency of alcohol consumption (per week)	0	214	126687.33	1.69 (1.47–1.92)	311	263379.75	1.18 (1.05–1.31)	1.43 (1.20–1.70)
	1–2	106	132751.18	0.80 (0.65–0.96)	165	278435.7	0.59 (0.50–0.69)	1.35 (1.06–1.72)
	≥3	32	39778.96	0.80 (0.53–1.11)	57	76272.11	0.75 (0.56–0.94)	1.08 (0.70–1.66)
BMI (kg/m^2)	<18.5	21	14073.79	1.49 (0.92–2.13)	25	24445.71	1.02 (0.65–1.43)	1.46 (0.82–2.61)
	18.5 to <25	397	286095.39	1.39 (1.25–1.53)	577	556513.37	1.04 (0.95–1.12)	1.34 (1.18–1.52)
	≥25	250	161880.9	1.54 (1.35–1.74)	392	343578.09	1.14 (1.03–1.25)	1.35 (1.16–1.59)
Total cholesterol (mg/dL)	<200	348	191136.9	1.82 (1.63–2.01)	491	378408.59	1.30 (1.18–1.41)	1.40 (1.22–1.61)
	≥200	192	162098.31	1.18 (1.02–1.36)	298	308647.36	0.97 (0.86–1.08)	1.23 (1.02–1.47)
Income	Low	263	184330.36	1.43 (1.26–1.60)	360	367543.17	0.98 (0.88–1.08)	1.46 (1.24–1.71)
	High	387	263349.28	1.47 (1.33–1.62)	600	526368.16	1.14 (1.05–1.23)	1.29 (1.13–1.46)
**(B) Outcome - Overall Parkinson’s disease related disorders**								
		**Case cohort**			**Reference cohort**			

		(n =112,554)			(n = 225,108)			IRR (95% CI)
		**Cases**	**Person-years**	**IR per 1000 person-years (95% CI)**	**Cases**	**Person-years**	**IR per 1000 person-years (95% CI)**	
All		926	460294.47	2.01 (1.88–2.14)	1378	921011.34	1.50 (1.42–1.58)	1.34 (1.24–1.46)
Age (years)	<60	313	354026.89	0.88 (0.79–0.98)	501	707785.04	0.71 (0.65–0.77)	1.25 (1.08–1.44)
	≥60	613	106267.58	5.77 (5.32–6.23)	877	213226.3	4.11 (3.84–4.39)	1.40 (1.26–1.55)
Sex	Male	426	211593.99	2.01 (1.82–2.21)	595	423267.94	1.41 (1.29–1.52)	1.43 (1.26–1.62)
	Female	500	248700.48	2.01 (1.84–2.19)	783	497743.4	1.57 (1.46–1.68)	1.28 (1.14–1.43)
Sex & Age group60 (years)	Male, <60	130	164389.96	0.79 (0.66–0.93)	213	328517.16	0.65 (0.56–0.74)	1.22 (0.98–1.52)
	Male, ≥60	296	47204.03	6.27 (5.57–6.99)	382	94750.78	4.03 (3.63–4.44)	1.56 (1.34–1.81)
	Female, <60	183	189636.93	0.97 (0.83–1.11)	288	379267.88	0.76 (0.67–0.85)	1.27 (1.06–1.53)
	Female, ≥60	317	59063.55	5.37 (4.79–5.96)	495	118475.52	4.18 (3.82–4.55)	1.28 (1.12–1.48)
Smoking status	Yes	91	74155.67	1.23 (0.98–1.48)	147	160454.62	0.92 (0.77–1.07)	1.34 (1.03–1.74)
	Ex-Smoking	676	303239.32	2.23 (2.06–2.40)	1017	607425.84	1.67 (1.57–1.78)	1.33 (1.21–1.47)
	No	159	82744.51	1.92 (1.63–2.22)	214	152580.4	1.40 (1.22–1.59)	1.37 (1.12–1.68)
Frequency of alcohol consumption (per week)	0	289	126192.46	2.29 (2.03–2.56)	450	260326.9	1.73 (1.57–1.89)	1.32 (1.14–1.54)
	1–2	153	132471.73	1.15 (0.97–1.34)	227	277440.87	0.82 (0.71–0.93)	1.41 (1.15–1.73)
	≥3	57	39667.19	1.44 (1.08–1.82)	73	76222.89	0.96 (0.75–1.18)	1.50 (1.06–2.12)

BMI (kg/m^2)	<18.5	28	14004.28	2.00 (1.29–2.78)	54	25552.33	2.11 (1.57–2.70)	0.95 (0.60–1.49)
	18.5 to <25	549	285055.56	1.93 (1.77–2.09)	753	551792.68	1.36 (1.27–1.46)	1.41 (1.26–1.58)
	≥25	348	161122.01	2.16 (1.94–2.39)	570	343379.88	1.66 (1.53–1.80)	1.30 (1.14–1.49)
Total cholesterol (mg/dL)	<200	470	190208.79	2.47 (2.25–2.70)	663	375639.81	1.76 (1.63–1.90)	1.40 (1.24–1.58)
	≥200	273	161508.19	1.69 (1.49–1.89)	433	307854.39	1.41 (1.28–1.54)	1.20 (1.03–1.40)
Income	Low	370	183470.02	2.02 (1.82–2.22)	546	366112.02	1.49 (1.37–1.62)	1.35 (1.19–1.54)
	High	525	262396.81	2.00 (1.83–2.17)	786	524944.83	1.50 (1.39–1.60)	1.34 (1.20–1.49)

Table S7. Cox proportional hazards analysis for the association between IBS (irritable bowel syndrome) and risk of Parkinson's disease.

HR, hazard ratio; CI, confidence interval.

[a] Adjusted for age and sex

[b] Adjusted for age, sex, smoking status, and alcohol consumption

[c] Adjusted for age, sex, smoking status, alcohol consumption, body mass index, total cholesterol and income

		**HR (95% CI)**			
		**Unadjusted**	**Model 1[a]**	**Model 2[b]**	**Model 3[c]**
All		1.24 (1.14–1.36)	1.24 (1.14–1.36)	1.31 (1.16–1.48)	1.38 (1.18–1.62)
Age (years)	<60	1.19 (0.93–1.53)	1.19 (0.93–1.53)	1.14 (0.81–1.59)	1.24 (0.83–1.84)
	≥60	1.12 (0.97–1.30)	1.12 (0.97–1.30)	1.24 (1.01–1.53)	0.97 (0.73–1.28)
Sex	Male	1.08 (0.90–1.31)	1.09 (0.91–1.31)	1.11 (0.87–1.40)	0.87 (0.65–1.17)
	Female	1.18 (0.99–1.40)	1.18 (1.00–1.41)	1.42 (1.08–1.85)	1.53 (1.06–2.19)
Smoking status	Yes	1.30 (0.84–2.00)	1.33 (0.86–2.06)	1.66 (0.96–2.87)	1.32 (0.70–2.52)
	No	1.09 (0.94–1.27)	1.10 (0.95–1.28)	1.24 (1.00–1.54)	1.13 (0.84–1.51)
	Ex-Smoking	1.18 (0.87–1.59)	1.22 (0.90–1.64)	1.06 (0.73–1.53)	0.91 (0.58–1.42)
Frequency of alcohol consumption (per week)	0	1.21 (0.96–1.51)	1.22 (0.97–1.53)	1.22 (0.97–1.53)	1.12 (0.82–1.52)
	1–2	1.15 (0.81–1.64)	1.19 (0.84–1.70)	1.20 (0.84–1.71)	1.04 (0.68–1.59)
	≥3	1.15 (0.71–1.87)	1.36 (0.84–2.22)	1.34 (0.83–2.19)	1.03 (0.60–1.78)
Total cholesterol (mg/dL)	<200	1.04 (0.87–1.24)	1.03 (0.87–1.23)	1.00 (0.76–1.33)	0.99 (0.74–1.32)
	≥200	1.07 (0.85–1.36)	1.13 (0.89–1.43)	1.29 (0.89–1.88)	1.25 (0.86–1.83)
Income	Low	1.21 (0.98–1.50)	1.25 (1.01–1.55)	1.38 (1.02–1.87)	1.26 (0.85–1.86)
	High	1.09 (0.93–1.28)	1.08 (0.92–1.27)	1.14 (0.91–1.42)	0.99 (0.75–1.32)
BMI (kg/m^2)	<18.5	1.06 (0.51–2.21)	1.09 (0.53–2.27)	1.57 (0.60–4.16)	1.66 (0.54–5.12)
	18.5 to <25	1.05 (0.89–1.24)	1.04 (0.88–1.23)	1.10 (0.87–1.40)	0.93 (0.68–1.26)
	≥25	1.29 (1.06–1.58)	1.32 (1.08–1.62)	1.42 (1.07–1.88)	1.29 (0.90–1.84)

Table S8. Cox proportional hazards analysis of the association between IBS (irritable bowel syndrome), with or without diarrhea, and the risk of Parkinson's disease.

HR, hazard ratio; CI, confidence interval.

[a] Adjusted for age and sex

[b] Adjusted for age, sex, smoking status, and alcohol consumption

[c] Adjusted for age, sex, smoking status, alcohol consumption, body mass index, total cholesterol and income

1. Exposure - IBS

		**HR (95% CI)**			
		**Unadjusted**	**Model 1** [a]	**Model 2** [b]	**Model 3** [c]
All		1.33 (1.24–1.43)	1.33 (1.24–1.44)	1.52 (1.37–1.68)	1.61 (1.41–1.85)
Age (years)	<60	1.30 (1.15–1.47)	1.30 (1.15–1.47)	1.47 (1.24–1.73)	1.53 (1.23–1.89)
	≥60	1.35 (1.23–1.48)	1.35 (1.23–1.48)	1.52 (1.33–1.74)	1.61 (1.36–1.92)
Sex	Male	1.34 (1.20–1.50)	1.35 (1.20–1.50)	1.48 (1.29–1.71)	1.54 (1.30–1.83)
	Female	1.32 (1.20–1.46)	1.32 (1.20–1.46)	1.56 (1.33–1.83)	1.74 (1.40–2.16)
Smoking status	Yes	1.41 (1.12–1.78)	1.51 (1.20–1.91)	1.69 (1.28–2.23)	1.83 (1.31–2.56)
	No	1.29 (1.19–1.41)	1.29 (1.18–1.41)	1.46 (1.28–1.67)	1.55 (1.30–1.85)
	Ex-Smoking	1.40 (1.16–1.67)	1.42 (1.18–1.70)	1.56 (1.26–1.94)	1.64 (1.27–2.12)
Frequency of alcohol consumption (per week)	0	1.49 (1.30–1.70)	1.52 (1.33–1.74)	1.52 (1.33–1.74)	1.61 (1.34–1.94)
	1–2	1.57 (1.29–1.90)	1.59 (1.32–1.93)	1.60 (1.32–1.94)	1.76 (1.39–2.23)
	≥3	1.16 (0.86–1.58)	1.35 (0.99–1.84)	1.33 (0.98–1.81)	1.34 (0.94–1.92)
Total cholesterol (mg/dL)	<200	1.38 (1.24–1.53)	1.36 (1.23–1.51)	1.69 (1.43–2.00)	1.69 (1.43–2.00)
	≥200	1.21 (1.06–1.38)	1.26 (1.10–1.44)	1.48 (1.19–1.85)	1.49 (1.19–1.86)
Income	Low	1.27 (1.13–1.43)	1.33 (1.18–1.49)	1.53 (1.29–1.81)	1.66 (1.33–2.06)
	High	1.37 (1.24–1.51)	1.34 (1.21–1.47)	1.50 (1.31–1.72)	1.58 (1.33–1.88)
BMI (kg/m^2)	<18.5	1.02 (0.67–1.56)	1.08 (0.71–1.64)	1.31 (0.71–2.41)	1.18 (0.49–2.86)
	18.5 to <25	1.31 (1.19–1.45)	1.31 (1.19–1.44)	1.60 (1.39–1.83)	1.67 (1.40–1.99)
	≥25	1.39 (1.23–1.57)	1.39 (1.24–1.57)	1.42 (1.20–1.68)	1.56 (1.26–1.93)

2. Exposure - IBS with diarrhea

**(A) Outcome - Parkinson's disease, idiopathic**					
		**HR (95% CI)**			
		**Unadjusted**	**Model 1** [a]	**Model 2** [b]	**Model 3** [c]
All		1.13 (1.00–1.29)	1.14 (1.00–1.29)	1.23 (1.03–1.47)	1.08 (0.86–1.35)
Age (years)	<60	1.19 (0.93–1.53)	1.19 (0.93–1.53)	1.14 (0.81–1.59)	1.24 (0.83–1.84)

	≥60	1.12 (0.97–1.30)	1.12 (0.97–1.30)	1.24 (1.01–1.53)	0.97 (0.73–1.28)
Sex	Male	1.08 (0.90–1.31)	1.09 (0.91–1.31)	1.11 (0.87–1.40)	0.87 (0.65–1.17)
	Female	1.18 (0.99–1.40)	1.18 (1.00–1.41)	1.42 (1.08–1.85)	1.53 (1.06–2.19)
Smoking status	Yes	1.30 (0.84–2.00)	1.33 (0.86–2.06)	1.66 (0.96–2.87)	1.32 (0.70–2.52)
	No	1.09 (0.94–1.27)	1.10 (0.95–1.28)	1.24 (1.00–1.54)	1.13 (0.84–1.51)
	Ex-Smoking	1.18 (0.87–1.59)	1.22 (0.90–1.64)	1.06 (0.73–1.53)	0.91 (0.58–1.42)
Frequency of alcohol consumption (per week)	0	1.21 (0.96–1.51)	1.22 (0.97–1.53)	1.22 (0.97–1.53)	1.12 (0.82–1.52)
	1–2	1.15 (0.81–1.64)	1.19 (0.84–1.70)	1.20 (0.84–1.71)	1.04 (0.68–1.59)
	≥3	1.15 (0.71–1.87)	1.36 (0.84–2.22)	1.34 (0.83–2.19)	1.03 (0.60–1.78)
Total cholesterol (mg/dL)	<200	1.04 (0.87–1.24)	1.03 (0.87–1.23)	1.00 (0.76–1.33)	0.99 (0.74–1.32)
	≥200	1.07 (0.85–1.36)	1.13 (0.89–1.43)	1.29 (0.89–1.88)	1.25 (0.86–1.83)
Income	Low	1.21 (0.98–1.50)	1.25 (1.01–1.55)	1.38 (1.02–1.87)	1.26 (0.85–1.86)
	High	1.09 (0.93–1.28)	1.08 (0.92–1.27)	1.14 (0.91–1.42)	0.99 (0.75–1.32)
BMI (kg/m^2)	<18.5	1.06 (0.51–2.21)	1.09 (0.53–2.27)	1.57 (0.60–4.16)	1.66 (0.54–5.12)
	18.5 to <25	1.05 (0.89–1.24)	1.04 (0.88–1.23)	1.10 (0.87–1.40)	0.93 (0.68–1.26)
	≥25	1.29 (1.06–1.58)	1.32 (1.08–1.62)	1.42 (1.07–1.88)	1.29 (0.90–1.84)
**(B) Outcome - Overall Parkinson's disease related disorders**					
		**HR (95% CI)**			
		**Unadjusted**	**Model 1 [a]**	**Model 2 [b]**	**Model 3 [c]**
All		1.31 (1.18–1.46)	1.32 (1.19–1.47)	1.43 (1.24–1.66)	1.37 (1.13–1.65)
Age (years)	<60	1.37 (1.14–1.65)	1.37 (1.14–1.65)	1.40 (1.09–1.79)	1.49 (1.10–2.01)
	≥60	1.29 (1.14–1.47)	1.29 (1.14–1.47)	1.42 (1.19–1.70)	1.25 (0.99–1.59)
Sex	Male	1.19 (1.01–1.38)	1.19 (1.02–1.39)	1.34 (1.10–1.63)	1.14 (0.90–1.45)
	Female	1.43 (1.24–1.66)	1.44 (1.25–1.66)	1.56 (1.25–1.94)	1.86 (1.37–2.51)
Smoking status	Yes	1.53 (1.10–2.12)	1.58 (1.14–2.18)	1.61 (1.08–2.41)	1.59 (1.00–2.55)
	No	1.30 (1.15–1.47)	1.30 (1.15–1.47)	1.46 (1.21–1.76)	1.47 (1.15–1.88)
	Ex-Smoking	1.22 (0.95–1.56)	1.26 (0.98–1.61)	1.28 (0.95–1.72)	1.10 (0.77–1.57)
Frequency of alcohol consumption (per week)	0	1.43 (1.18–1.73)	1.46 (1.21–1.77)	1.46 (1.21–1.77)	1.43 (1.10–1.86)
	1–2	1.40 (1.07–1.83)	1.45 (1.11–1.89)	1.45 (1.11–1.90)	1.51 (1.10–2.07)
	≥3	1.10 (0.72–1.69)	1.25 (0.82–1.92)	1.27 (0.82–1.94)	0.91 (0.54–1.51)
Total cholesterol (mg/dL)	<200	1.18 (1.02–1.37)	1.18 (1.02–1.37)	1.25 (0.99–1.57)	1.25 (0.99–1.58)
	≥200	1.37 (1.13–1.66)	1.43 (1.18–1.73)	1.59 (1.18–2.16)	1.61 (1.18–2.19)
Income	Low	1.31 (1.11–1.56)	1.35 (1.14–1.60)	1.32 (1.05–1.67)	1.39 (1.03–1.88)
	High	1.31 (1.14–1.50)	1.30 (1.13–1.49)	1.52 (1.26–1.84)	1.34 (1.06–1.71)

BMI (kg/m²)	<18.5	1.64 (0.84–3.22)	1.63 (0.83–3.19)	1.42 (0.61–3.30)	1.32 (0.48–3.63)
	18.5 to <25	1.25 (1.09–1.43)	1.24 (1.08–1.42)	1.35 (1.12–1.63)	1.21 (0.95–1.55)
	≥25	1.42 (1.19–1.68)	1.45 (1.22–1.72)	1.58 (1.25–2.00)	1.66 (1.23–2.25)

2. IBS without diarrhea

**(A) Outcome - Parkinson's disease, idiopathic**					
		**HR (95% CI)**			
		**Unadjusted**	**Model 1** [a]	**Model 2** [b]	**Model 3** [c]
All		1.34 (1.22–1.48)	1.35 (1.22–1.49)	1.40 (1.22–1.60)	1.42 (1.19–1.69)
Age (years)	<60	1.45 (1.21–1.73)	1.45 (1.21–1.73)	1.38 (1.11–1.72)	1.26 (0.94–1.68)
	≥60	1.31 (1.16–1.47)	1.31 (1.16–1.47)	1.38 (1.17–1.64)	1.46 (1.18–1.81)
Sex	Male	1.38 (1.19–1.59)	1.38 (1.19–1.60)	1.45 (1.21–1.73)	1.31 (1.05–1.63)
	Female	1.32 (1.15–1.50)	1.32 (1.15–1.51)	1.34 (1.09–1.64)	1.64 (1.23–2.19)
Smoking status	Yes	1.73 (1.23–2.42)	1.90 (1.36–2.67)	1.94 (1.31–2.87)	1.64 (1.00–2.67)
	No	1.34 (1.19–1.50)	1.33 (1.19–1.49)	1.41 (1.19–1.67)	1.52 (1.21–1.90)
	Ex-Smoking	1.17 (0.92–1.48)	1.19 (0.94–1.51)	1.17 (0.89–1.54)	1.18 (0.86–1.62)
Frequency of alcohol consumption (per week)	0	1.43 (1.20–1.70)	1.47 (1.23–1.74)	1.46 (1.23–1.74)	1.69 (1.35–2.13)
	1–2	1.35 (1.06–1.72)	1.33 (1.04–1.70)	1.32 (1.03–1.69)	1.15 (0.84–1.57)
	≥3	1.08 (0.70–1.66)	1.23 (0.80–1.90)	1.22 (0.79–1.88)	1.03 (0.62–1.69)
Total cholesterol (mg/dL)	<200	1.40 (1.22–1.61)	1.40 (1.22–1.60)	1.48 (1.20–1.83)	1.48 (1.19–1.83)
	≥200	1.23 (1.02–1.47)	1.27 (1.06–1.52)	1.35 (1.01–1.81)	1.32 (0.98–1.77)
Income	Low	1.46 (1.24–1.71)	1.53 (1.31–1.80)	1.51 (1.22–1.87)	1.39 (1.04–1.84)
	High	1.29 (1.13–1.46)	1.25 (1.10–1.42)	1.31 (1.10–1.57)	1.43 (1.15–1.78)
BMI (kg/m²)	<18.5	1.46 (0.82–2.61)	1.63 (0.91–2.92)	1.46 (0.65–3.29)	0.81 (0.25–2.64)
	18.5 to <25	1.34 (1.18–1.52)	1.34 (1.18–1.52)	1.50 (1.26–1.80)	1.54 (1.22–1.93)
	≥25	1.35 (1.16–1.59)	1.35 (1.15–1.58)	1.26 (1.02–1.56)	1.30 (1.00–1.71)
**(B) Outcome - Overall Parkinson's disease related disorders**					
		**HR (95% CI)**			
		**Unadjusted**	**Model 1** [a]	**Model 2** [b]	**Model 3** [c]
All		1.34 (1.24–1.46)	1.35 (1.24–1.46)	1.41 (1.25–1.57)	1.42 (1.23–1.64)
Age (years)	<60	1.25 (1.08–1.44)	1.25 (1.08–1.44)	1.33 (1.12–1.59)	1.19 (0.95–1.49)
	≥60	1.40 (1.26–1.55)	1.40 (1.27–1.55)	1.43 (1.24–1.66)	1.55 (1.28–1.87)
Sex	Male	1.43 (1.26–1.62)	1.43 (1.27–1.62)	1.49 (1.28–1.74)	1.44 (1.20–1.74)
	Female	1.28 (1.14–1.43)	1.28 (1.14–1.43)	1.30 (1.10–1.54)	1.38 (1.10–1.74)

Smoking status	Yes	1.34 (1.03–1.74)	1.44 (1.11–1.87)	1.56 (1.15–2.12)	1.44 (0.99–2.09)
	No	1.33 (1.21–1.47)	1.32 (1.20–1.45)	1.35 (1.17–1.56)	1.38 (1.14–1.66)
	Ex-Smoking	1.37 (1.12–1.68)	1.41 (1.15–1.74)	1.47 (1.15–1.87)	1.52 (1.14–2.03)
Frequency of alcohol consumption (per week)	0	1.32 (1.14–1.53)	1.35 (1.16–1.56)	1.35 (1.17–1.57)	1.45 (1.19–1.77)
	1–2	1.41 (1.15–1.73)	1.40 (1.14–1.72)	1.40 (1.14–1.72)	1.30 (1.01–1.68)
	≥3	1.50 (1.06–2.13)	1.73 (1.23–2.46)	1.73 (1.22–2.46)	1.57 (1.05–2.34)
Total cholesterol (mg/dL)	<200	1.40 (1.25–1.58)	1.38 (1.23–1.55)	1.47 (1.23–1.75)	1.47 (1.23–1.76)
	≥200	1.20 (1.03–1.40)	1.26 (1.08–1.46)	1.31 (1.04–1.67)	1.33 (1.04–1.70)
Income	Low	1.35 (1.19–1.54)	1.42 (1.24–1.62)	1.52 (1.27–1.82)	1.67 (1.32–2.11)
	High	1.34 (1.20–1.49)	1.30 (1.17–1.45)	1.32 (1.13–1.53)	1.28 (1.06–1.54)
BMI (kg/m^2)	<18.5	0.95 (0.60–1.50)	1.04 (0.66–1.65)	1.03 (0.55–1.96)	0.66 (0.24–1.78)
	18.5 to <25	1.41 (1.26–1.58)	1.40 (1.26–1.57)	1.49 (1.28–1.73)	1.53 (1.26–1.86)
	≥25	1.30 (1.14–1.49)	1.30 (1.14–1.49)	1.33 (1.10–1.59)	1.35 (1.08–1.69)

Figure S1. Kaplan–Meier curve for outcome-free survival following IBS (irritable bowel syndrome) diagnosis.

1. IBS

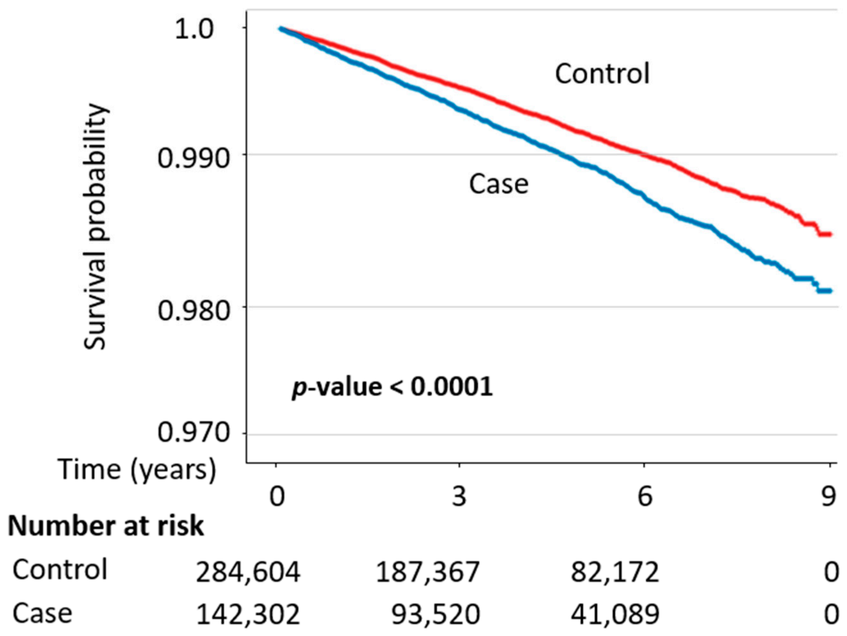

2. IBS with diarrhea

(a) Outcome - Parkinson’s disease, idiopathic

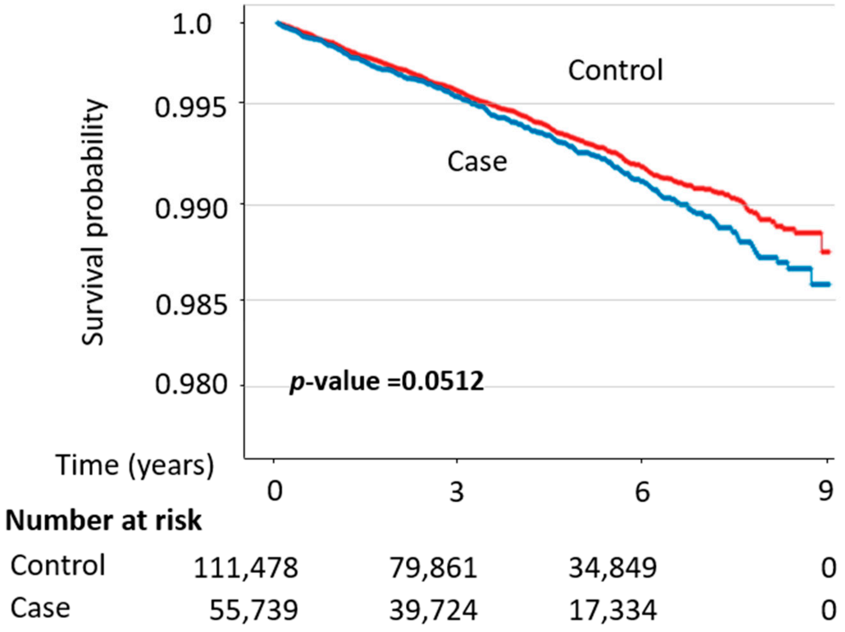

(b) Outcome - Overall Parkinson’s disease related disorders

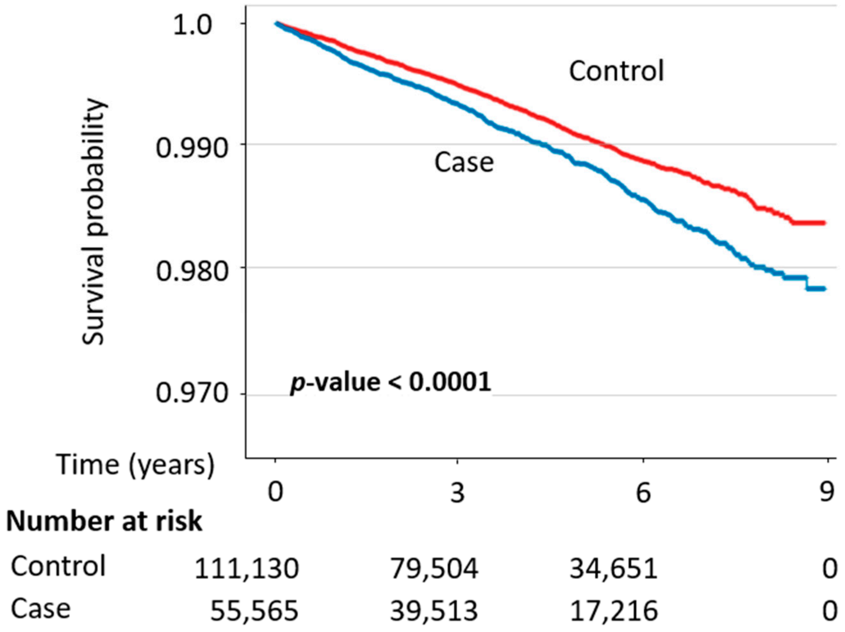

3. IBS without diarrhea

(a) Outcome - Parkinson’s disease, idiopathic

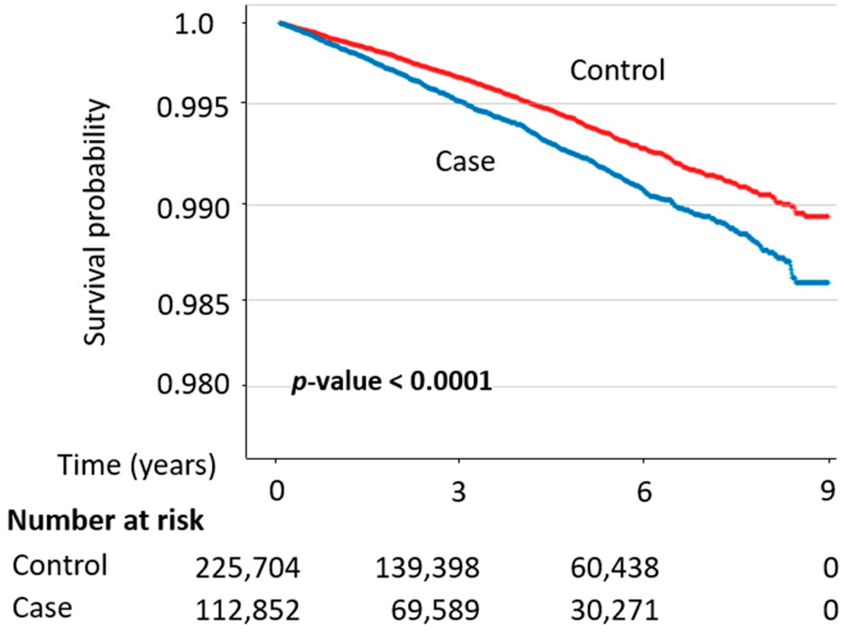

(b) Outcome - Overall Parkinson’s disease related disorders

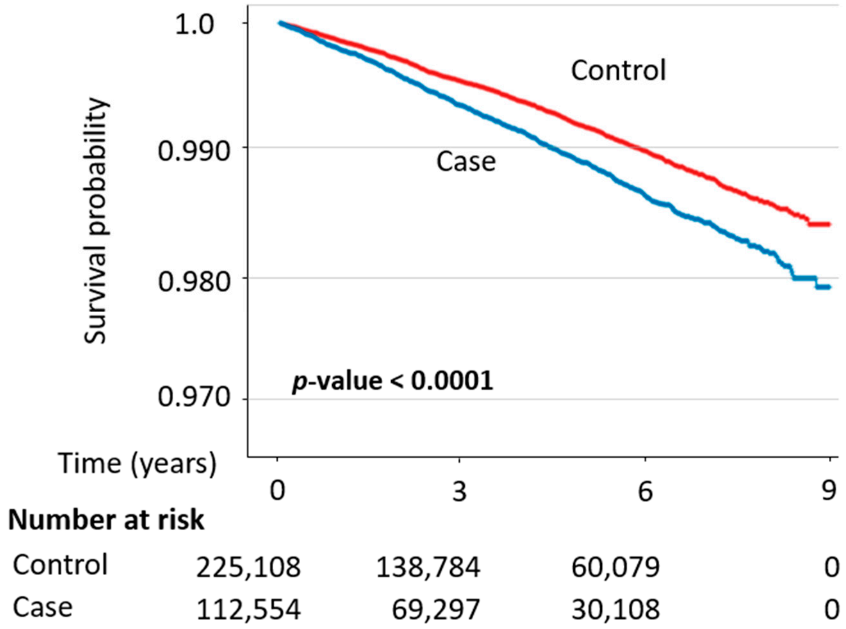

Figure S2. Forest plot of adjusted hazard ratios (aHRs) with 95% confidence intervals for the risk of outcome among individuals with IBS (irritable bowel syndrome), stratified by sex and age.

CI, confidence interval; aHR, adjusted hazard ratio.

1. IBS

Outcome - Overall Parkinson's disease related disorders

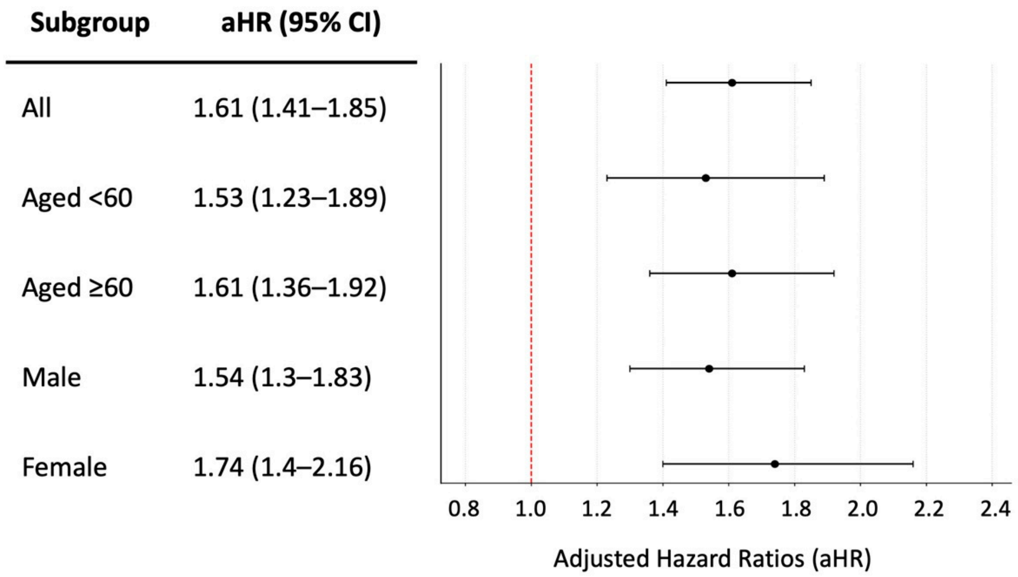

2. IBS with diarrhea

Outcome A - Parkinson's disease, idiopathic

Outcome B - Overall Parkinson's disease related disorders

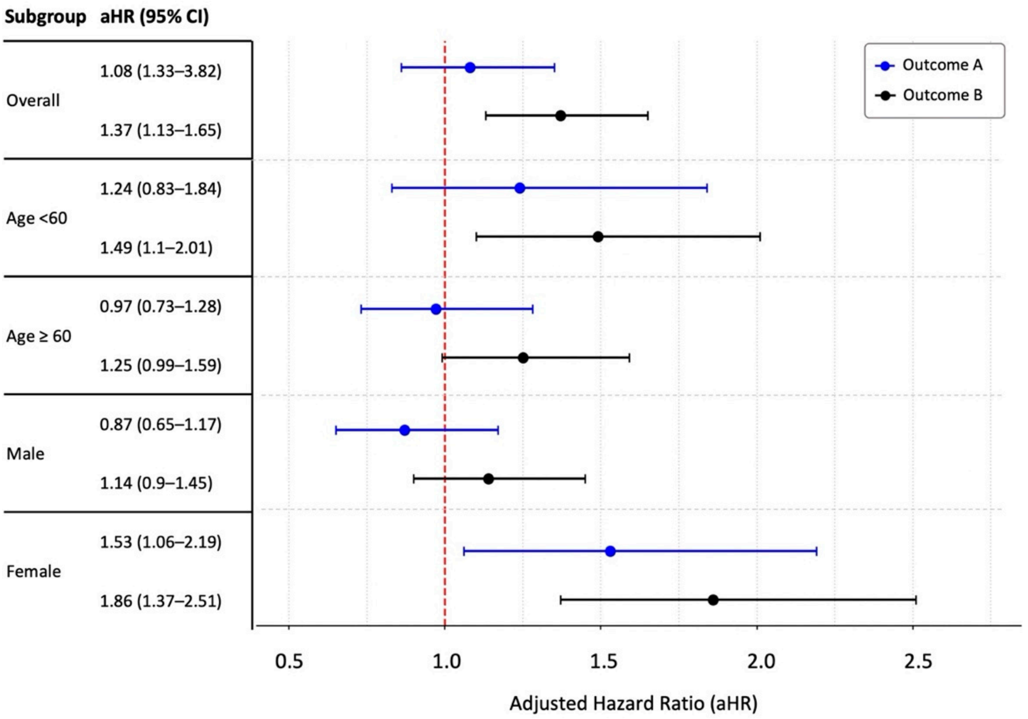

3. IBS without diarrhea

Outcome A - Parkinson's disease, idiopathic

Outcome B - Overall Parkinson's disease related disorders

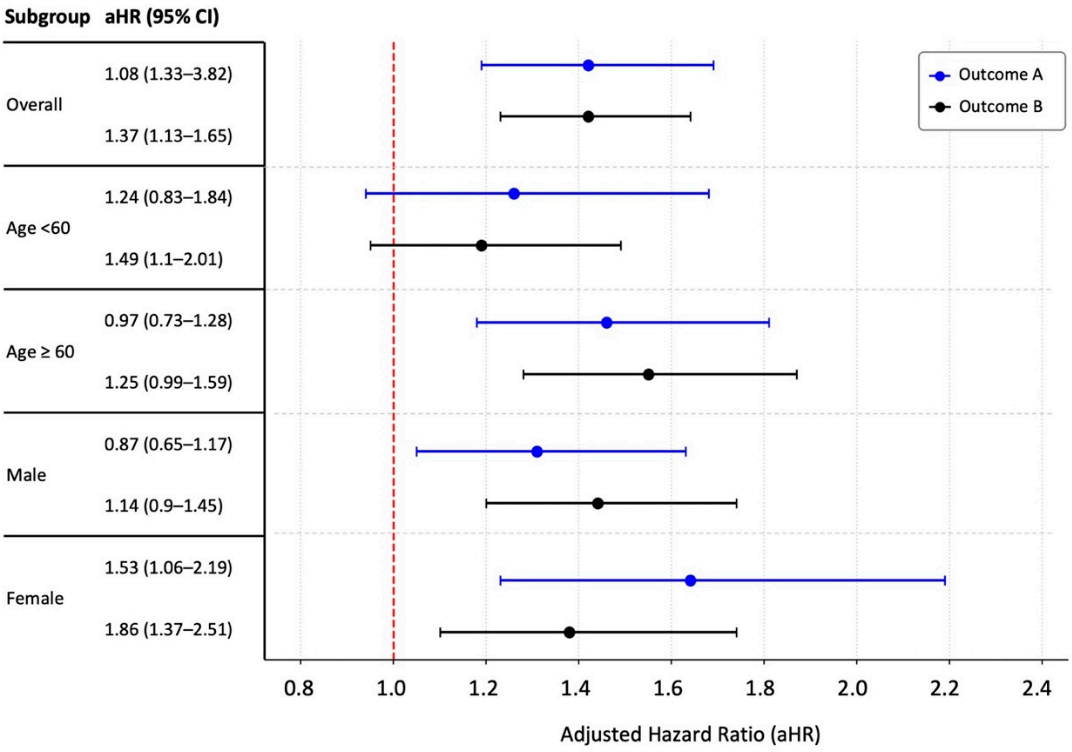

Supplement: Supplementary file 1 [file healthcare-14-01329-s001.zip › healthcare-4278811-supplementary.pdf]
